# A comprehensive review on probiotics and their use in aquaculture: Biological control, efficacy, and safety through the genomics and wet methods

**DOI:** 10.1016/j.heliyon.2024.e40892

**Published:** 2024-12-04

**Authors:** Matteo Calcagnile, Salvatore Maurizio Tredici, Pietro Alifano

**Affiliations:** Department of Experimental Medicine, University of Salento, Lecce, Italy

**Keywords:** Probiotics, Aquaculture, Genomics, Safety, Wet methods

## Abstract

Probiotics, defined as viable microorganisms that enhance host health when consumed through the diet, exert their effects through mechanisms such as strengthening the immune system, enhancing resistance to infectious diseases, and improving tolerance to stressful conditions. Driven by a growing market, research on probiotics in aquaculture is a burgeoning field. However, the identification of new probiotics presents a complex challenge, necessitating careful consideration of both the safety and efficacy of the microorganisms employed. This review aims to delineate the most utilized and effective methods for identifying probiotics. The most effective approach currently combines in silico analysis of genomic sequences with in vitro and in vivo experiments. Two main categories of genetic traits are analyzed using bioinformatic tools: those that could harm the host or humans (e.g., toxin production, antibiotic resistance) and those that offer benefits (e.g., production of helpful compounds, and enzymes). Similarly, in vitro experiments allow us to examine the safety of a probiotic but also its effectiveness (e.g., ability to adhere to epithelia). Finally, in vivo experiments allow us to study the effect of probiotics on fish growth and health, including the ability of the probiotic to manipulate the host's microbiota and the ability to mitigate the infections. This review comprehensively analyzes these diverse aspects, with a particular focus on the potential of studying the interaction between bacterial pathogens and probiotics through these integrated methods.

## Highlights

• Bioinformatic identify positive or negative traits by studying the probiotic genome.
• In vitro experiments allow the efficacy and safety of probiotics to be evaluated.
• In vivo experiments evaluate the safety and ability of probiotics to improve health.
• Bioinformatics, and in vitro/in vivo experiments can be integrated into a pipeline.
• By integrating these methods, the biocontrol capacity of probiotics is highlighted.

## Graphical abstract

**Figure:**
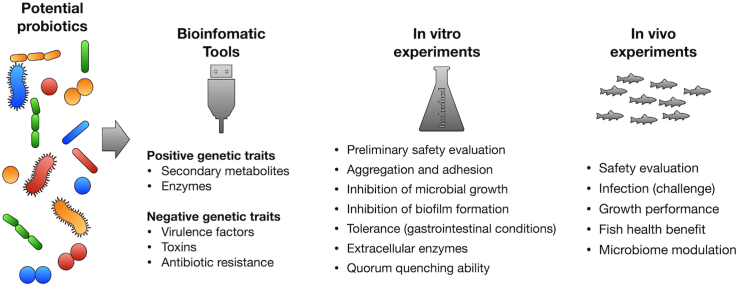

## 1 Introduction

More than a hundred years ago, scientist Metchnikoff theorized that human health and the effects of aging could be influenced by manipulating the intestinal microbiota thanks to the beneficial bacteria in yogurt [1]. The term probiotic was used for the first time by Lilly and Stillwell [2] who called with this name one or more substances produced by a protozoan, and which stimulated the growth of another protozoan. Subsequently, probiotics were referred to as “live micro-organisms which when administered in adequate amounts confer a health benefit on the host” [3].

Probiotics can be classified based on the type of microorganism (bacteria vs. non-bacteria), on the ability to form spores (sporeforming vs. non-sporeforming), on the composition in terms of types of microorganisms of the product (multi-species/-strains probiotics vs single-species/-strain probiotics), or on the presence of the same microorganism in the normal microflora of the host (allochthonous probiotics vs autochthonous probiotics) [4]. The most widespread probiotics are Gram-positive bacteria belonging to the *Lactobacillus*, *Bacillus,* and *Bifidobacterium* genera although there are probiotic formulations based on Gram-negative bacteria (*Escherichia coli*) and fungi (*Saccharomyces*) [4].

The functions attributed to probiotics and related to gut health are multiple and include increasing digestion and absorption of nutrients, improving the intestinal barrier, modulating the immune system, and promoting healthy bacterial flora [[4], [5], [6], [7]]. Probiotics can regulate host innate and adaptive immune responses by modulating the functions of dendritic cells, macrophages, and T and B lymphocytes [8]. In addition, probiotics can regulate signaling pathways, such as the NF-kB pathway involved in the inflammatory response [9]. Furthermore, microorganisms used as probiotics can produce antimicrobial substances, such as bacteriocins [10], which act on potentially pathogenic microorganisms that can cause disease in the host.

In addition to gut health, probiotics have also been studied in various contexts such as oral cavity microbiome [11], vaginal microbiome [12], and allergy prevention [13]. Probiotics have also been studied for the mitigation of intestinal disorders caused by celiac disease [14].

Microorganisms used as probiotics have been isolated from various sources, including fermented and non-animal food products [15]. Other probiotics have been isolated directly from the intestinal tract of humans or other animals as well as from milk. Additionally, some probiotics have been isolated from environmental samples, such as soil, sediment, plants, and water [[16], [17], [18]].

In addition to improving the health and well-being of humans, probiotics are increasingly used in different sectors such as agriculture and animal breeding. Probiotics used in agriculture can act directly on plants by stimulating their growth and can improve the absorption of nutrients, such as phosphates [19,20]. Furthermore, they can act indirectly on the health of the plant, inhibiting the growth of phytopathogenic microorganisms or modulating the composting of phytophagous insects [18,21,22].

Additionally, prebiotics—non-digestible food ingredients—can modulate the composition of the gastrointestinal microbiota and promote the production of beneficial metabolites [23]. Among these metabolites, short-chain fatty acids (SCFAs) are important. They are derived from the fermentation of complex carbohydrates and exert beneficial effects through simple diffusion and specific receptors found only in the epithelial cells of higher vertebrates. Consequently, commensal gut microbes can be modulated through the dietary administration of target microbes, non-digestible compounds, or a combination of both [23]. Both strategies have demonstrated the ability to provide resistance against pathogens, mitigate stress effects, and restore homeostasis [23].

As with probiotics administered to humans, probiotics for animal nutrition contribute to the health and well-being of animals. Probiotics alter the intestinal microbiota of animals and reduce the spread of pathogens by reducing the need for antibiotic administration to animals [24]. The bacteria most used as probiotics in livestock belong to the genus *Lactobacillus* and *Bifidobacteria*. The consumption of probiotics has been associated with a health improvement but also with an increase in animal productivity [4,25,26].

In aquaculture probiotics are used to promote the health and growth of aquatic organisms [[27], [28], [29]]. Even in fish, probiotics have been shown to inhibit the growth of harmful pathogens, improve disease resistance, improve nutrient digestibility, and increase stress tolerance.

The application of probiotics in aquaculture is a rapidly expanding sector. The global market size is valued at over $190 million in 2022 and is expected to reach $285 million in 2028 (https://www.360marketupdates.com/global-aquaculture-probiotics-market-24105409). As the aquaculture probiotics market grows, more and more researchers are engaged in the isolation and characterization of new strains that can be used as food additives in aquaculture.

This review aims to illustrate and discuss the most employed and effective methods for identifying, characterizing, and testing potential probiotics using in silico, in vitro, and in vivo methodologies. Identifying a new probiotic for aquaculture applications is a great and interesting challenge that includes several activities. Among these activities, it is important to develop rapid screening methods that aim to identify the genetic traits important for a probiotic which include both nutritional aspects, such as the production of extracellular enzymes, and aspects linked to the ability of probiotics to fight infections and stress in the host organism improving its health. In addition to this, it is also important to highlight genetic traits that are potentially dangerous for human and animal health. Among these traits, the presence of toxins, virulence factors, and genes that determine resistance to antibiotics can be mentioned.

Furthermore, the reduction in the cost of sequencing methods together with the development of bioinformatics tools now allows these approaches to be used to characterize probiotics. Genomic methods are useful to identify positive and negative genetic traits of microorganisms. At the same time, host microbial communities can be studied in greater detail using metagenomic methods which have the advantage of also highlighting non-culturable microorganisms.

## 2 Features of probiotics in the genomic era

The first bacterium whose genome was fully sequenced was *Haemophilus influenzae* in 1995 [30]. Since then, Whole Genome Sequencing (WGS) has been extensively employed on both pathogenic and beneficial microorganisms. The development of this branch of 'omics' sciences can be primarily attributed to two main factors: 1) the reduction in sequencing costs and 2) the advancement, dissemination, and enhancement of computing, encompassing both hardware and software, which are valuable for conducting bioinformatics analyses. When it comes to reducing sequencing costs, the data is very clear: the cost per megabase (Mb) of sequencing has decreased from over $5000 to $0.006 in approximately 20 years (from 2001 to 2021) [31].

The following delves into genomics, encompassing bioinformatic analysis, and the utilization of these tools for the identification and study of probiotics. Several studies including genomic and transcriptomic data were analyzed with the objectives of identifying and describing the most used methods to characterize the genome of a potential probiotic (Table S1).

### 2.1 Genomic of probiotics: assessing the key genetic trait of a probiotic

**Fig. 1:**
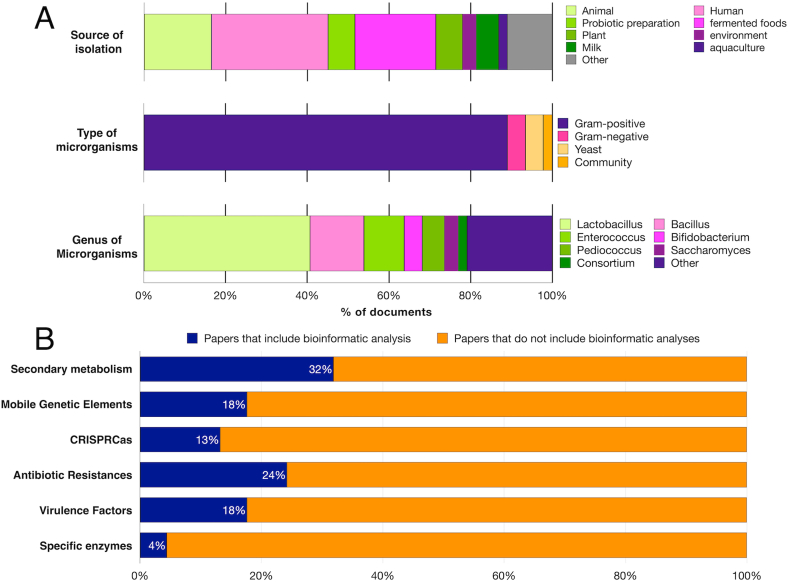
Types of probiotics analyzed using WGS and bioinformatic tools. A) Microorganisms clustered by source of isolation (above), type of microorganisms (middle), and genus (below). **B)** Bioinformatic tools used for genome analysis and the percentage of documents analyzed that included a specific bioinformatic analysis.

An extensive literature search was conducted, encompassing studies published between 2011 and 2023, which focused on probiotics applied to human or animal nutrition (Table S1). Most of the studies concerned Gram-positive probiotics (89 %) and single microorganisms (88 %) (Fig. 1A). On the other hand, only 4 % of the papers concern Gram-negative bacteria and another 4 % concern yeast (Fig. 1A).

Among the probiotics most studied in this type of analysis are bacteria of the genus *Lactobacillus* (40 %), with detailed reference to the *L. fermentum* (6 documents) [[32], [33], [34], [35], [36], [37]], *L. plantarum* (8 documents) [[38], [39], [40], [41], [42], [43], [44], [45]], *L. rhamnosus* (5 documents) [[46], [47], [48], [49], [50]], and *L. salivarius* (3 documents) [[51], [52], [53]] species (Fig. 1A–Table S1). In addition, 15 documents deal with other species belonging to the *Lactobacillus* genus [[54], [55], [56], [57], [58], [59], [60], [61], [62], [63], [64], [65], [66], [67], [68]]. Other kinds of batteries studied in these documents were the bacteria of the *Bacillus* (13 %) and *Enterococcus* (10 %) genera (Fig. 1A–Table S1). Among the *Bacillus* genus, the most studied bacteria were *B. coagulans* (3 %) [[69], [70], [71]] and *B. subtilis* (3 %) [[72], [73], [74]], while among the *Enterococcus* genus the most studied species were *E. faecalis* (3 %) [75,[76], [77]] and *E. faecium* (4 %) [[78], [79], [80], [81]].

In addition, 7 documents deal with other species belonging to the *Bacillus* genus [[82], [83], [84], [85], [86], [87]] and 3 to the *Enterococcus* genus [88,89]. Other genera of bacteria included in the studies were *Bifidobacterium* (4 %) [[90], [91], [92], [93]] and *Pediococcus* (6 %) [[94], [95], [96], [97], [98]] (Fig. 1A–Table S1). Three documents concerned *Saccharomyces boulardii* [[99], [100], [101]], while two concerned probiotic consortia [102,103] (Fig. 1A–Table S1). Other genera of potential probiotics primarily characterized using WGS and bioinformatics were: *Lactococcus* [104], *Weissella* [105], *Streptomyces* [106] *Paenibacillus* [107,108], *Shewanella* [109], *Clostridium* [110], *Schleiferilactobacillus* [111], *Leuconostoc* [112] *Weizmannia* [113], *Propionibacterium* [114], *Escherichia* [115], *Vibrio* [116], *Streptococcus* [117], *Tetragenococcus* [118], *Sporolactobacillus* [119], *Burkholderia* [120], *Lysinibacillus* [121], and the yeast *Kluyveromyces* (Quarella et al., 2016).

Most of the probiotics studied in these papers were isolated from human samples (29 %), animal samples (16 %), or fermented food (20 %). The probiotics were isolated from plants or probiotic preparations in 6 studies, from environmental samples in 3 studies, from milk in 5 studies, and aquaculture samples in 2 studies (Fig. 1A–Table S1).

The bioinformatics tools employed in the articles of Table S1 were discussed in the subsequent section.

### 2.2 Computational tools employed to identify probiotic traits from genomic sequences

Bioinformatics tools are among the simplest methods for identifying potential probiotics by analyzing genomic sequences. Typically, these tools analyze the genome to identify genes and proteins and subsequently deduce whether the microorganism possesses specific characteristics. These characteristics can be either positive (e.g., secondary metabolism) or negative (e.g., virulence, antibiotic resistance). Researchers often utilize automatic annotation systems such as RAST (Rapid *Annotation* using Subsystem Technology) [122], PROKKA (suitable for annotating de novo assemblies) [123], PANZER2 [124] and KAAS (KEGG Automatic Annotation Server) [125]. Other annotation systems involve the use of databases such as COG (Clusters of Orthologous Groups) [126]. These systems are employed to identify the specific function of proteins and then assess whether a gene exhibits positive, negative, or neutral traits for potential probiotic applications. The use of automatic annotation systems plays a crucial role in genome mining as it streamlines the annotation of all Coding Sequences (CDS) and proteins within the genome. These systems often provide information about both their general functions (e.g., metabolism) and specific functions (e.g., enzyme activity).

However, searching for specific functions using only the general annotation has some disadvantages such as the attribution of different functions to a single gene and the functional prediction of the gene, regardless of the genomic context. For these reasons, specific bioinformatic tools are employed to predict the characteristics of microorganisms, including secondary metabolites, virulence factors, antibiotic resistance genes, mobile genetic elements, CRISPR-Cas systems, and specific enzymes (Table 1).

**Table 1:** Common bioinformatics tools used to analyze the genome of potential probiotics.

	Tools	Ref.
**Secondary metabolism**	SMIPS	[127]
	NRPSpredictor	[128]
	antiSMASH	[129]
	BAGEL	[130], [131]
**Mobile Genetic Elements**	PlasmidFinder	[132]
	PHASTER	[133]
	PHAST	[134]
	PATRIC website	[135]
	ISfinder	[136]
	Prophage Hunter	[137]
	ISsaga	[138]
	OASIS	[139]
**CRISPRCas**	CRISPRTarget	[140]
	CRISPRFinder	[141]
	CRISPRDetect	[142]
**Virulence Factors**	VirulenceFinder	[143]
	PathogenFinder	[144]
	VFDB	[145]
	Nullarbor	[146]
	MiFuP Safety	https://www.nite.go.jp/nbrc/mifup/#
	IslandViewer 4	[147]
	PATRIC website	[135]
**Specific enzymesand proteins**	MEROPS	[148]
	CAZy database	[149]
	CAZymes analysis toolkit (CAT)	[150]
	ResFinder	[151]
	CARD	[152]
	Nullarbor	[146]
	MiFuP Safety	https://www.nite.go.jp/nbrc/mifup/#
	ARDB	[153]

**Secondary metabolism.** Secondary metabolites have different effects on the host such as stimulation of the immune system or the biological control of pathogens. Furthermore, some of these metabolites act as toxins. Several bioinformatics tools based on genomics are valuable for predicting the biosynthesis of secondary metabolites. Approximately 32 % of the studies listed in Table S1 employ one or more of these bioinformatics tools to identify genes associated with secondary metabolite biosynthesis (Fig. 1B). The most frequently used tool is antiSMASH [129], followed by BAGEL [130,131], SMIPS [127] and NRPSpredictor [128].

**Virulence Factors.** Safety is a central aspect to investigate before administering a probiotic. At the genomic level, it is important to define the presence of virulence factors. Numerous virulence factors have been discovered in bacteria, and these factors play a crucial role in helping pathogens invade the host organism and evade the host's immune defenses. Among the various virulence factors are enzymes (such as collagenase, protease, elastase, and neuraminidase), and toxins. Notably, some microorganisms like pathogenic clostridia (*Clostridium botulinum* and *Clostridium tetani*) release disease-causing toxins known as exotoxins. In contrast, Gram-negative bacteria possess lipopolysaccharides (LPS), referred to as endotoxins, which are constituents of their bacterial cell walls. Consequently, most probiotics have been identified within the Gram-positive group, which includes genera such as *Bacillus*, *Lactobacillus*, and *Bifidobacterium*. However, it's essential to acknowledge that potentially beneficial bacteria may also possess some virulence factors. Therefore, it is of fundamental importance to sequence the genome of potential probiotics and subsequently identify the presence of genes responsible for known virulence factors. Approximately 18 % of the studies listed in Table S1 employ one or more bioinformatics tools or databases intending to identify these virulence factors (Fig. 1B).

The most frequently used database for identifying virulence factors is the Virulence Factor Database (VFDB) [145]. Two other frequently used tools are PathogenFinder and VirulenceFinder [144,143]. Additional tools used for this purpose include Nullarbor [146], Microbial Functional Potential (MiFuP) (https://www.nite.go.jp/nbrc/mifup/#), and IslandViewer 4 [147].

**Antibiotic Resistances.** Antibiotic resistance is a critical focus of contemporary microbiological research. Antibiotic resistance genes (ARGs) naturally occur in bacteria and can be transferred from one bacterium to another. The overuse and improper use of antibiotics have contributed to the evolution and widespread dissemination of ARGs, posing a significant threat to public health. Considering that probiotics are live microorganisms consumed for their health benefits, ensuring their safety, and minimizing risks to human health is paramount. ARGs in probiotics raise concerns about the potential transfer of ARGs to pathogenic bacteria, rendering them more challenging to treat with antibiotics.

In approximately 24 % of the documents listed in Table S1, researchers employ one or more bioinformatics tools or databases to identify ARGs (Fig. 1B). The most frequently used databases for this purpose are ARDB (Antibiotic Resistance Genes Database) and CARD (The Comprehensive Antibiotic Resistance Database), each cited in 6 documents [153,152]. CARD encompasses all the data found in ARDB. Access to this database is facilitated through the Resistance Gene Identifier (RGI) tool, available both online and as a stand-alone application. RGI is referenced in 4 documents included in Table S1. MiFuP Safety and Nullarbor, as mentioned earlier in the section discussing virulence factors, are two additional tools utilized for identifying ARGs. Finally, ResFinder is a freely accessible tool that aids users in analyzing Whole-Genome Sequencing (WGS) results to pinpoint antimicrobial resistance determinants (AMR) [151].

**Mobile Genetic Elements.** Mobile Genetic Elements (MGEs) encompass genetic material capable of mobility within the same genome or transfer to other microorganisms. These elements include plasmids, transposons, integrons, introns (group I and II), and prophages. CRISPR systems, although often associated with MGEs, will be discussed separately. In typical bacterial genomes, a collection of MGEs, collectively referred to as the 'mobilome', is present. Identifying and sequencing these elements is crucial as they can carry genes for antibiotic resistance and other traits.

In approximately 18 % of the studies listed in Table S1, researchers utilize one or more bioinformatics tools or databases to identify MGEs (Fig. 1B). The bioinformatics tools used for this research are as follows: PlasmidFinder [132], PHAST (PHAge Search Tool) and PHASTER [134,133], Prophage Hunter [137], IS Finder (Insertion Sequence Finder) [136], Insertion Sequence Semi-Automatic Genome Annotation (ISsaga) [138], PATRIC website [135] and the Optimized Annotation System for Insertion Sequences (OASIS) [139] (Table 1). In addition, sequence alignment algorithms can be used in a customized way. For example, a BLAST search was executed against a specialized plasmid database accessible on the PATRIC website [135] to identify MGEs. Similarly, BLASTn search was executed against the *Saccharomyces* Genome Database (SGD) using the *S. boulardii* genome as input, specifically targeting retrotransposon sequences and the results were validated manually [99,101,154].

**CRISPR-Cas.** The CRISPR-Cas system is a defense mechanism found in bacteria that offers acquired immunity against foreign genetic elements like viruses or plasmids. It consists of short, highly conserved DNA repeats separated by variable sequences known as spacers, often situated near *cas* gene. The presence of CRISPR-Cas loci contributes to the genomic stability of bacterial strains, enabling them to adapt to diverse environments. This system has the potential to prevent probiotics from acquiring virulence or antibiotic-resistance genes through horizontal gene transfer, a crucial aspect of ensuring their safety and effectiveness as therapeutic agents. To identify these elements within genome sequences, various tools are employed (Table 1, Fig. 1B), including CRISPRTarget [140], CRISPRFinder [141], and CRISPRDetect [142].

**Enzymes.** In addition to ensuring probiotic safety, bioinformatic methods were utilized to identify specific types of enzymes that equip the bacterium with the biochemical capabilities to metabolize substrates [[150], [148], [149]]. These tools are instrumental in the identification of Carbohydrate-Active Enzymes (CAZymes), which are enzymes capable of both constructing and breaking down complex carbohydrates and glycoconjugates [155]. Furthermore, these tools can be used to pinpoint proteases and proteolytic enzymes. Such information serves various purposes, including the design of tailored diets for a specific probiotic and enhancing our understanding of the bacterium's role during the digestive and assimilation processes within the host organism (Table S1, Fig. 1B).

## 3 Features of probiotics – wet assays

**Fig. 2:**
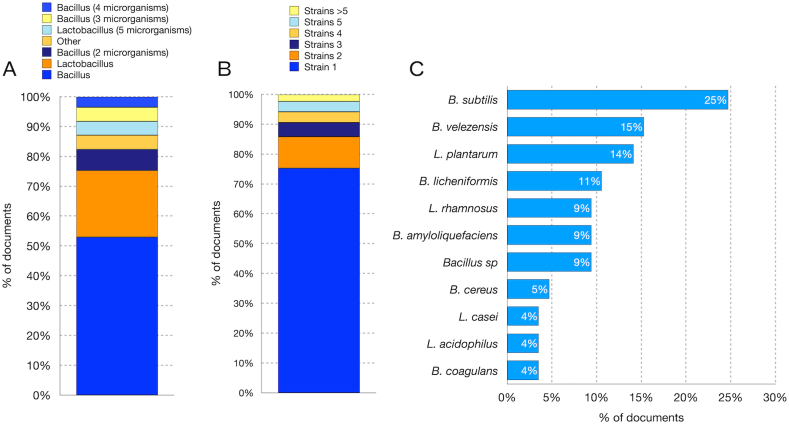
Type of probiotics characterized using in vitro or in vivo methods. **A)** Genus of probiotics analyzed. **B)** Number of probiotics analyzed simultaneously in the same paper. **C)** Species most used in in vitro characterization experiments or in vivo administration.

Most of the documents in the previous section concerned probiotics of the *Bacillus* or *Lactobacillus* genus. Consequently, only documents relating to these bacterial genera will be discussed in this second section. Therefore, in this section, a further 89 documents were analyzed which concerned the use of *Lactobacillus* and *Bacillus* strains in aquaculture. Most of the studies analyzed and reported in Table S2 concern probiotics of the *Bacillus* genus (53 %) compared to bacteria of the *Lactobacillus* genus (22 %) (Fig. 2A). In some cases, multiple species or different strains of *Bacillus* were used (Fig. 2A and B): 2 microorganisms [156,157,[157], [158], [159], [160]], 3 microorganisms[[161], [162], [163], [164]], 4 microrganisms [[165], [166], [167]]. Likewise, in some cases multiple species or multiple strains of *Lactobacillus* have been used [[168], [169], [170], [171]]. In other cases, a species of *Bacillus* has been used in combination with probiotics belonging to other genera such as *Lactococcus* and *Paenibacillus* [172,173] (Fig. 2A and B).

Among the *Bacillus* species, the most extensively studied were *B. subtilis* [[174], [175], [176], [177], [178], [179], [180], [181], [182], [183], [184]], *B. velezensis* [[184], [185], [186], [187], [188], [189], [190], [191], [192], [193]], *B. amyloliquefaciens* [[194], [195], [196], [197], [198], [199]], *B. licheniformis* (11 %) [[200], [201], [202], [203]], *B. cereus* [[7], [204], [205], [206]], *B. coagulans* [[207], [208], [209]] or other *Bacillus* species [[210], [211], [212], [213], [214]] (Fig. 2C).

Within the *Lactobacillus* genus, the most researched species included *L. plantarum* (Van Doan et al., 2021a; [[215], [216], [217], [218], [219], [220]]), *L. rhamnosus* [[221], [222], [223], [224], [225], [226], [227]], *L. casei* [228,229], *L. acidophilus* [230,231] (Fig. 2C). In addition, other species of *Lactobacillus* were used as probiotics, such as *L. reuteri* [232].

In studies involving two probiotics, the bacteria were administered separately to evaluate the effect of the single probiotic [156,172], or as a mix of two different bacteria [157,158,233]. In this last case, the mix included bacteria of the same genus [[156], [157], [158]] or a combination of *Bacillus* and *Lactobacillus* [172,233]. Furthermore, commercial probiotic preparations containing multiple species have also been the subject of studies involving the administration of fish [167].

In some cases, the same bacteria were first studied in vitro and subsequently administered as single strains or as a combination of strains (Kuebutornye et al., 2020a Kuebutornye et al., 2020b). Furthermore, several studies focused on the characterization of specific probiotics using in vitro methods [161,162,168,234]. Finally, it is noteworthy that some studies concern the administration together with probiotics of other additives such as propionic acid [157] and β-glucans [169,170,211].

In most cases (82 %), probiotics were administered through the diet. In some instances, probiotics were added to water (4 %) were injected intraperitoneally (5 %) (Table S2). The intraperitoneal injection was often used to assess the pathogenicity of the probiotics. Interestingly, probiotics were administered via a vector organism, *Moina micrura,* which was used to encapsulate the probiotic [212].

### 3.1 Source of isolation

**Fig. 3:**
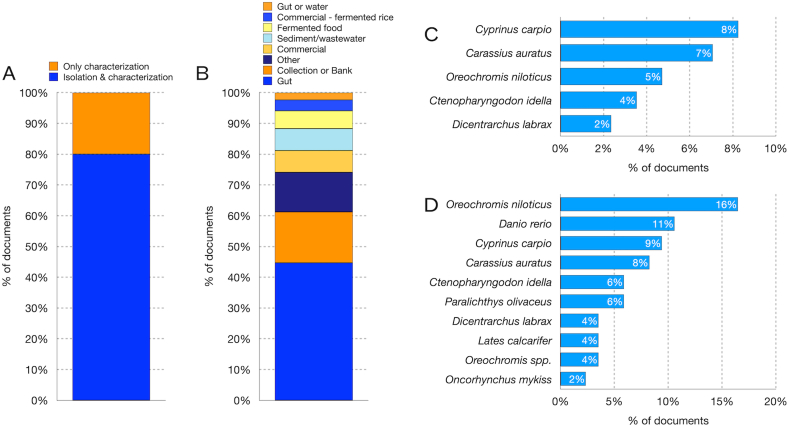
Types of samples from which the probiotic was isolated, and the fish used for in vivo testing. **A)** Papers that included probiotic isolation. **B)** Types of samples from which the probiotic was isolated. **C)** Fish species used to collect the sample from which the probiotic was isolated. **D)** Fish species used for in vivo testing.

Many probiotics used in aquaculture experiments were acquired from commercial strain banks or banks managed by research centers and universities (Fig. 3A and B), However, 17 out of 85 documents (20 %) included the isolation of the potential probiotic used in aquaculture experiments (Fig. 3A). In this last case, the samples used to isolate the bacteria were collected from aquaculture specimens, with a focus on fish samples, particularly from the intestines of various fish species, including *Carassius auratus, Ctenopharyngodon idella, Cyprinus carpio, Dicentrarchus labrax,* and *Oreochromis niloticus* (Fig. 3C).Other samples used to isolate a probiotic included water samples from fish farms and various types of water samples, such as natural waters, wastewater, soil, sediment, and fermented foods (Fig. 3B).

The most common types of fish from which probiotics were isolated were *Cyprinus carpio* (8 %), *Carassius auratus* (7 %), *Oreochromis niloticus* (5 %), *Ctenopharyngodon idella* (4 %), and *Dicentrarchus labrax* (2 %) (Fig. 3C). Often but not always the fish being tested belonged to the same species from which the probiotic was isolated. The most common fish species used to test the effect of the probiotic were: *Oreochromis niloticus* (16 %), *Danio rerio* (11 %), *Cyprinus carpio* (9 %), *Carassius auratus* (8 %), *Ctenopharyngodon idella* (6 %), *Paralichthys olivaceus* (6 %), *Dicentrarchus labrax* (6 %), *Lates calcarifer* (4 %), other *Oreochromis* spp. (2 %), and *Oncorhynchus mykiss* (2 %) (Fig. 3D).

### 3.2 In vitro characterization of probiotics

Certainly, in vitro experiments are valuable for analyzing various traits of probiotics. Here are some of the key traits that can be investigated through in vitro experiments.

i) Aggregation and adhesion properties. In vitro assays can assess the ability of probiotics to aggregate, adhere to surfaces, or interact with other microorganisms. These properties are important for understanding their potential to colonize and interact with the host.
ii) Inhibition of microbial growth and biofilm formation. Probiotics may possess antimicrobial properties that inhibit the growth of harmful microorganisms. They can also disrupt or prevent the formation of biofilms, which are often associated with pathogenicity.
iii) Tolerance to gastrointestinal conditions. Simulated gastrointestinal conditions can be created in the laboratory to evaluate how well probiotics survive and function in the harsh environment of the digestive tract. This helps determine their viability as oral supplements.
iv) Production of extracellular enzymes. Probiotics may produce extracellular enzymes that are active on protein or carbohydrate macromolecules. These enzymes can have various roles, including aiding in digestion and modulating the gut environment.

In vitro experiments provide controlled environments for studying these traits, allowing researchers to gain insights into the potential benefits of probiotics for fish health.

#### 3.2.1 Adherence ability of probiotics

The ability to adhere to epithelia or other biological surfaces is one of the most significant characteristics of probiotics, as it facilitates intestinal colonization and enhances the activity of the immune system. Typically, probiotics exhibit high aggregation capacities with each other and with other microorganisms, including fish pathogens. To assess this trait, several experimental procedures can be used, such as auto-aggregation, co-aggregation, cell surface hydrophobicity, and in vitro adhesion to artificial epithelium.

Auto-aggregation refers to the ability of probiotic cells to aggregate with each other. This ability was evaluated by measuring the rate at which probiotic cells settle at the bottom of a test tube or by using spectrophotometric methods to monitor changes in turbidity over time [161,163,168,185,210,235]. The auto-aggregation test involves growing the probiotic in broth, collecting it through centrifugation, and resuspending the bacteria in a saline solution, such as phosphate buffer (PBS). The solution is then incubated at 26–28 °C for a selected period without stirring. The absorbance (A) at 600 nm of the upper layer is measured both at the beginning (A_0_) of experiment and after the incubation period (A_1_). Auto-aggregation is calculated as follows:

Autoaggregation = 1− (A1/A0) × 100

Similarly, co-aggregation measures the ability of probiotic cells to aggregate with other microorganisms, including potential pathogens. This property can help probiotics prevent harmful microorganisms from adhering to host tissues [161,210]. The co-aggregation assay is conducted as follows: both the pathogenic bacterium and the probiotic are cultured; they are subsequently collected through centrifugation, and they are resuspended in PBS or other saline solutions. The A at 600 nm is then individually measured for each (A__probiotic_, A__pathogen_), and the two bacteria are subsequently combined in equal volumes and subjected to incubation at 26–28 °C for a selected period without stirring. After incubation, the optical density (A__mix_) of the upper layer of this solution is measured. Co-aggregation is calculated as follows:

Co-aggregation=((1− A_mix_)/ ((A_probiotic_ + A_pathogen_)/2)) × 100

Another experiment that is used frequently is the Cell Surface Hydrophobicity (CSH) assay [161,163,168,185,210,235]. This assay assesses the hydrophobic properties of probiotic cell surfaces. A more hydrophobic surface can enhance adhesion to hydrophobic biological surfaces. As with the other methods, the bacteria are grown, collected, and resuspended in a saline solution. For this experiment, Ringer's solution was typically used (NaCl 2.25 g/L, NaHCO_3_ 0.05 g/L, CaCl_2_ 0.12 g/L, KCl 0.105 g/L, pH 7.0 ± 0.2). At this stage, the initial A (A_0_) is measured. Subsequently, the bacteria are suspended in a solvent (either xylene or chloroform) and incubated. After incubation, the A is measured at 600 nm at the specified time points (A_1_). CHS is calculated as follows:

CHS= (1 − A_1_/A_0_) × 100

In addition, functionalized supports or artificial epithelia have been utilized to estimate adhesion capacities. Probiotic adhesion to these surfaces can be quantified, providing insights into their potential for colonizing the gut and exerting beneficial effects. Supports are functionalized using films of molecules derived from the intestine, which can either be pure (mucin binding assay) [210] or obtained directly from fish in the form of intestinal mucus (adhesion mucus assays) [190]. Artificial epithelia are cultured in vitro using either human cell lines, such as Caco-2 [214,235], or fish-specific lines, such as RTgutGC [156]. Various techniques have been employed to assess the adherence of the probiotic, including fluorescence microscopy or CFU counting. Using CFU count, adhesion is calculated as follows:

% Adhesion = 100 × (log CFU _bacteria adhered_ ÷ log CFU _bacteria added_)

The ability to form biofilms is a common trait among probiotics. Various methods have been employed to assess biofilm formation, including growth in Mueller Hinton agar supplemented with Congo red dye and culturing microorganisms in a liquid medium on artificial surfaces like slides [163,201,208]. In the latter case, the biomass of the biofilm is assessed using the crystal violet method [162,201,208]. Similarly, the capacity to form biofilms is also a crucial characteristic of many human and animal pathogens, often linked to antibiotic resistance [236]. Therefore, some studies incorporate anti-biofilm assays [162]. Antibiofilm activity assays can be designed as competition assays [162].

#### 3.2.2 Antimicrobial and quorum quenching potential assays

Among the typical traits of probiotics, one stands out: the ability to influence pathogenic microorganisms through various mechanisms, such as the synthesis of molecules with bactericidal properties or through nutritional competition. The simplest type of assay used for this purpose is the microbiological assay [[161], [162], [163],^166^,^168^,^179^,[185], [186], [187], [188], [189],^192^,^203^,^208^,^210^,^212^,^235^]. In this type of analysis, a tester microorganism is used as an indicator of the bactericidal action of the probiotic.

Typically, the tester organism is inoculated into a suitable culture medium containing a low percentage of agar (0.7 %). This medium is poured into a Petri dish, creating a layer around 2–3 mm thick [237]. Probiotics can be cultivated in both solid and liquid media. In the first case, an agar disc with a diameter of 1.5 cm is taken from the plate containing the probiotic and placed in the center of the prepared dish. In the second case, an aliquot of the exhausted liquid medium is taken and plated as a spot in the center of the prepared dish. This method is highly versatile, allowing for the use of various microorganisms, including both Gram-positive and Gram-negative bacteria. Tester bacteria can be broadly categorized into two types: reference bacteria or fish pathogens. Reference bacteria encompass: i) human pathogenic or opportunistic Gram-negative bacteria (such as *Salmonella enterica* and *Pseudomonas aeruginosa*). ii) human pathogenic or opportunistic Gram-positive bacteria (*Staphylococcus aureus*). iii) Gram-negative or Gram-positive bacteria commonly employed in laboratory settings, such as *Escherichia coli* or *Micrococcus luteus*. *M. luteus* is frequently used because it is sensitive to antimicrobial compounds and antimicrobial peptides (such as lysozyme), as described further below. These species offer the advantage of easy cultivation in laboratory conditions, and the results can be applicable in identifying valuable probiotics for human drug development. However, if the goal is to define probiotics as useful in aquaculture, it is advisable to conduct tests on fish pathogens. Fish pathogens include both Gram-negative bacteria (*Aeromonas hydrophila, Aeromonas veronii, Aeromonas schubertii, Vibrio anguillarum, Vibrio harveyi, Vibrio mimicus, Yersinia ruckeri*) and Gram-positive bacteria (*Streptococcus agalactiae, Streptococcus iniae, Edwardsiella tarda*). The effects and spread of these pathogens are discussed in the section related to challenge experiments (fish infections).

Other assays can be employed to comprehend the interaction between a probiotic strain and a pathogenic strain. For instance, the co-culture inhibition assay [179,190,238] is one such method. This test detects effects caused both by bacterial competition for the nutritional resources present in a medium and by the production of secondary metabolites with bactericidal or bacteriostatic effects [238]. The procedure involves inoculating an equal number of probiotic and pathogen cells into the culture medium. Subsequently, the culture is allowed to grow for a predetermined period, after which the suspension is plated to count viable bacteria. By counting colonies with different phenotypes or by using selective media, it is possible to determine the number of bacteria of both the probiotic and pathogen. By comparing these values to a control, it is possible to calculate the inhibition value.

The antibacterial activity was typically measured through direct analysis of the isolates. However, other methods can be employed to analyze the production of secondary metabolites in greater detail and ultimately identify and purify them [192,196]. For instance, an HPLC assay was used to identify and quantify macrolactin A produced by *B. amyloliquefaciens* X030 [196]. Furthermore, probiotic cultures can be used to obtain extracts that are enriched in the metabolites of interest. For example, the extract obtained from *B. velezensis* WLYS23, was used to perform MIC and experiment against Gram-positive and Gram-negative bacteria [192].

Another important aspect is the ability of probiotics to negatively modulate the signaling circuits of quorum sensing (QS) in pathogenic bacteria. Qs is a density-dependent regulatory system found in both Gram-negative and Gram-positive bacteria. QS plays a critical role in regulating virulence, biofilm formation, and various aspects of microorganism physiology. Consequently, it has become a target system for innovative therapies aimed at inhibiting QS [239]. The negative modulation potential of QS is referred to as the quorum quenching (QQ) potential and is assessed using both culture and molecular methods [162,203,234]. Culture methods typically employ a sensitive bacterium, such as luminescent bacteria (*Vibrio* spp.) or *Chromabacterium violaceum* ATCC12472. These bacteria either emit light or form pigmented colonies when QS is active. For this reason, these bacterial species are used as indicators to assess the QQ activity [162,203,234]. In addition, a mutant strain of *C. violaceum* (*C. violaceum* CV026), which does not produce pigment due to the absence of the QS signal, can be used to measure AHL-lactonase activity. In this case, the signal molecule (C6-HSL, N-Hexanoyl-L-homoserine lactone) is added to the medium inducing color development [162,203,234]. On the contrary, the AHL-lactonase activity leads to the degradation of the C6-HSL. In addition, molecular methods are employed to identify genes related to QQ activity [234].

#### 3.2.3 Tolerance and susceptibility assays

Probiotics carry out their function in the intestine, and as a result, they traverse the entire digestive system of the fish before colonizing the intestinal epithelium. However, it's important to note that it has been estimated that approximately 20 % of fish lack a true stomach [240]. Consequently, fish exhibit a wide diversity of digestive systems, adapted to their various feeding habits. Fish with a stomach typically maintain an acidic pH. For example, tilapia stomachs in the Salton Sea exhibit changes in acidity levels throughout different seasons, with pH values ranging from around 5.5 to values below 5 [241]. Furthermore, some bacteria are sensitive to bile salts, which adds another layer of complexity. Fish also have different types of bile salts, with this variability being more closely linked to the phylogenetic relationships among fish species rather than their diet [242].

Resistance to acidity can be assessed through simple culture tests in which the probiotic is cultivated in an appropriate medium, and the increase in biomass is evaluated using spectrophotometric measurements or by counting viable bacteria (CFU/mL) [161,185,189]. In other studies, acidity resistance is assessed by inoculating the bacteria in phosphate buffer (PBS) with varying pH levels and measuring CFU/mL [190]. Typically, a pH range spanning from acidic (2 or 3) to neutral (7) or alkaline (9) is used in these tests [161,185,189], although in some cases, only a single acidic pH value is chosen [190]. Incubation times range from a few hours (1.5) [190] to 12 or 24 h [161,189].

Experiments involving bile salts are conducted similarly, using different concentrations of bile salts (0.02 %, 0.04 %, 0.06 %, 0.08 %, 0.10 %) [189] or a single concentration (0.3 %) [161,168,201,214]. The increase in biomass is evaluated after a few hours. Commercial bile salt reagents from cattle or fresh bile extracted from the fish used in the experiment are generally employed for these tests [208]. In one of these cases, the percentage of bile used was higher than in previous studies, reaching up to 20 % [208].

In more complex experiments, the bacteria were inoculated under simulated gastrointestinal conditions. In one instance, the bacteria were exposed to artificial gastric juice for 2 h, which contained 2 g/L NaCl and 3.2 g/L pepsin powder, to determine live bacteria counts (CFU/mL) [214]. In other cases, alongside gastric juice, intestinal fluid was also simulated by modifying the growth conditions during incubation through the following steps: i) acidification (pH 2) and the addition of a porcine pepsin solution (0.04 g pepsin in 1 mL 0.1 M HCl) (3 h of incubation). ii) an increase in pH to 5.3 using 0.9 M NaHCO_3_, along with the addition of 200 μL of sodium glycodeoxycholate (0.15 g/mL), sodium taurodeoxycholate (0.1 g/mL), taurocholate (0.15 g/mL), and 100 μL of pancreatin (0.08 g/mL). iii) Basification (pH 8). The sample was incubated for 8 h to complete the in vitro digestion process, and the suspension was plated to count the CFU/mL [235]. In addition, the conditions of the stomach and intestines can also be simulated through separate experiments [210].

Other tolerance assays encompass high-temperature tolerance and sodium chloride tolerance [185]. Sodium chloride tolerance is particularly relevant when a probiotic is intended for use in marine fish, while high-temperature tolerance is essential in establishing a feed processing protocol.

Furthermore, assessing the susceptibility of bacteria to lysozyme could serve as a valuable method for selecting probiotics. For instance, it has been suggested that lysozyme in breast milk can favor certain types of bifidobacteria while limiting others, thus promoting the development of healthy microbiota in human infants [243]. Similarly, lysozyme resistance may enhance colonization of the fish gut, making high lysozyme tolerance an important criterion for selecting superior probiotics [185].

Finally, the resistance of probiotic bacteria to heavy metals, such as cadmium [205,209], is especially useful when these bacteria are employed to mitigate and counteract symptoms associated with heavy metal poisoning in fish. To assess bacterial sensitivity to heavy metals, the most effective methods are the determination of the minimum inhibitory concentration (MIC) followed by the minimum bactericidal concentration (MBC) [205]. Other heavy metal resistance tests measure the absorption of metals by bacteria [209].

#### 3.2.4 Extracellular enzymes

Other critical characteristics of a probiotic pertain to its ability to secrete extracellular enzymes, which may confer benefits to the host during digestion. Some of these enzymes include amylase, protease, lipase, chitinase, gelatinase, and cellulase. The production of these enzymes is evaluated using appropriate solid media containing colloidal solutions or indicators (Table 2). For instance, a modified version of the Luria-Bertani medium containing nonfat-dried milk (1.5 %) or a specific medium like Skim Milk Agar proves useful in assessing extracellular protease production.

**Table 2:** Assays for extracellular enzymes secreted by probiotics belong to the *Bacillus* and *Lactobacillus* genera: functional solid medium and quantitative methods.

Medium/buffer	Composition	Active component	Enzyme and Assay Type	Ref
Nutrient Agar (modified)	Tryptone 10 g/L NaCl 5 g/L Beef extract 3 g/L Agar 15 g/L	Nonfat-dried milk (0.5–1%)	Proteases Halo-assay (colloidal suspension)	Wu et al., 2021 [179] [201]
		Starch, soluble (0.2–1%)	Amylase Halo-assay (after coloration with iodine solution)	
		CMC (0.5–1%)	Cellulase Halo-assay (after coloration with Congo red solution)	
		Xylan 1 %	Xylanase Halo-assay (after coloration with Congo red solution)	
		Gelatin	Gelatinase activity, Halo-assay (after coloration with 15 % HgCl2)	
		Tributyrin	Lipase Halo-assay (precipitated fatty acids)	
LB Agar (modified)	Tryptone 10 g/L NaCl 10 g/L Yeast extract 5 g/L Agar 15 g/L	Nonfat-dried milk (1–1.5 %)	Proteases Halo-assay (colloidal suspension)	[192] [201]
		Starch, soluble (1–1.5 %)	Amylase Halo-assay (after coloration with iodine solution)	
		CMC (1 %)	Cellulase Halo-assay	
		Triglyceride tributyrate (1 %)	Lipase Halo-assay	
Skim Milk Agar	Casein peptone 5 g/L Yeast extract 2.5 g/L Skim milk 1 g/L Glucose 1 g/L Agar 10.5 g/L pH 7	nonfat-dried milk (1.5 %)	Proteases Halo-assay (colloidal suspension)	[161]
Starch Agar	Meat Extract 3 g/L Peptic digest 5 g/L Agar 15 g/L pH 7.2	Starch, soluble 2 g/L	Amylase Halo-assay (after coloration with iodine solution)	[161]
Tween Agar	Solution A: Peptone 10 g NaCl 5.0 g CaCl_2_ 2H_2_O 0.1 g H_2_O 900 mL Solution B: Tween 80 10 g H_2_O 900 mL Agar 15 g/L		Lipase Halo-assay (after coloration with iodine solution)	[161]
phenolphthalein phosphate agar	Peptic digest 5 g/L Beef extract 3 g/L NaCl 5 g/L Agar 15 g/L pH 7.4 ± 0.2	Sodium phenolphthalein phosphate 0.012 g/L	Phosphatase (pink colour formation)	[179]
Christensen Urea Agar	Peptone 1 g/L Dextrose 1 g/L NaCl 5 g/L Na_2_HPO 1.2 g/L KH_2_PO_4_ 0.8 g/L Agar 15 g/L pH 6.8 ± 0.2	50 ml of sterile 40 % Urea Solution Phenol red 0.012 g/L	Urease (pink colour formation)	[179]
Spirit blue agar	Casein 10 g/L Yeast Extract 5 g/L Agar 17 g/L pH 6.8 ± 0.2	Tributyrin (2 %, v/v) Tween 80 (0.3 %, v/v) Spirit Blue 0.15 g/L	Lipase	[201]
Quantitative methods using a buffer	Borate 10 mmol/L pH 8.0 50 mM glycine NaOH pH 9.0 PBS 0.1M pH 6.8 PBS 20 mM 10 mM NaCl 6.9	Casein suspension (2 %)	Proteases Absorbance (275–280 nm)	[210] Mohapatra et al., 2003 [166] Rick & Stegbauer, 1974.
		Casein suspension (2 %)	Proteases Absorbance (680 nm, after treatment with Na_2_CO_3_ and Folin-Phenol)	
		CMC (1 %)	Cellulase Absorbance (574 nm)	
		Starch, soluble (1 %)	Amylase Absorbance (540–546 nm, after treatment with DNS^a^)	

a Dinitrosalicylic acid reagent (1 % (w/v) 3,5-dinitrosalicylic acid, 30 % (w/v) potassium-sodium tartrate).

The presence of extracellular enzymes can be assessed both qualitatively and quantitatively [208] (Table 2). Among the reporter molecules, dinitrosalicylic acid can be employed to quantify amylase activity, casein to measure protease activity, Tween to gauge lipase activity, and carboxymethylcellulose to evaluate cellulase activity [208] (Table 2).

### 3.3 Safety evaluation of probiotics

One of the most crucial aspects in the development of new probiotics is assessing their safety profile. In this regard, it's important to note that certain bacteria exhibit Pathogen-Associated Molecular Patterns (PAMPs) that function by activating innate immunity through Pattern Recognition Receptors (PRRs), including Toll-like receptors (TLRs). These PAMPs encompass various types of molecules, such as double-stranded RNA, glycoproteins containing mannose as a terminal residue that is absent in mammals, and lipopolysaccharide (LPS). The latter molecule is present in the cell walls of Gram-negative bacteria, which are generally less suitable for use as probiotics. This suggests that even bacteria initially categorized as non-pathogenic have the potential to activate both the innate and adaptive immune responses. To assess the safety of a probiotic, both in vitro approaches, typically employed as preliminary assays, and in vivo experiments can be utilized.

#### 3.3.1 In vitro safety

Several in vitro assays are employed to assess the safety of using a bacterium as a probiotic, including hemolytic activity, antibiotic susceptibility tests, toxin detection, and cytotoxicity assays.

Hemolytic activity refers to a microorganism's ability to cause the breakdown of red blood cells, and it's a critical safety consideration for probiotics. Hemolytic activity can potentially lead to various health problems, such as anemia, sepsis, or other serious conditions. Hemolytic activity can be assessed in several ways, with one common method being the blood agar plate assay [158,161,163,179,185,188,189,192,201,214,235]. In this assay, a bacterial strain is streaked onto an agar plate containing red blood cells. If the bacteria exhibit hemolytic activity, a clear zone will be visible around the bacterial colonies where the blood has been lysed. Other methods for assessing hemolytic activity include spectrophotometry [244].

As previously mentioned, it's crucial for probiotics not to contain antibiotic resistance genes (AGRs) to prevent contributing to antibiotic resistance spread. Consequently, testing for antibiotic resistance plays a pivotal role in evaluating the safety profile of probiotics [4]. This type of analysis can be conducted in two ways: the antibiogram in a solid medium and the MIC in culture broth [245]. In addition, molecular-based assays using PCR are versatile and can serve various purposes. For instance, PCR can be utilized to detect AGRs or genes encoding toxins. In a study on *Bacillus* sp. KRF-7 employed as a probiotic for rockfish, the presence of certain genes linked to the bacterium's virulence was examined, including hemolysin (*hbl*), several enterotoxins (*nheB, bceT, entFM*), cytotoxin K (*cytK*), sphingomyelinase (*sph*), and phospholipase C (*plc*) [214].

One effective method for assessing bacterial cytotoxicity in vitro is to co-culture potential probiotics with cell cultures. For this purpose, models of human intestinal epithelium (Caco-2) and specific cell lines isolated from fish, such as fibroblast cell lines from *Cyprinus carpio* [179], were utilized. For this type of assay, a bacterial strain is incubated with the epithelium, and the cells are examined to determine if the bacteria have induced any damage or cytotoxic effects, such as cell death or alterations in cell morphology. Common metrics for assessing cytotoxicity include lactate dehydrogenase release assays and the MTT [3-(4,5-Dimethylthiazol-2-yl)-2,5-Diphenyltetrazolium Bromide] assay. Caco-2 cells can also be utilized to assess the probiotic's ability to adhere to the intestinal epithelium [214,235].

#### 3.3.2 In vivo safety

The safety of a fish probiotic can be assessed in vivo through various pathogenicity assays. In an in vivo pathogenicity assay, fish are exposed to the potential probiotic through injection, immersion in contaminated water, or oral administration. The fish are monitored for the development of clinical symptoms and mortality. Different types of organisms can be used for this type of test, including aquatic and non-aquatic. For example, the toxicity of *B. licheniformis* T-1 was evaluated in vivo using *Chlorella vulgaris*, *Daphnia magna*, and *Brachionus calyciflorus* [203]. These organisms are typically utilized in toxicology and serve as environmental indicators to determine the presence of hazardous contaminants in environmental matrices. These organisms offer several advantages: they are easy to cultivate in the laboratory, require minimal breeding time and costs, and provide diverse types of information, including insights into the probiotic's safety as an environmental contaminant. However, a notable drawback is their substantial biological differences from fish, resulting in limited information transferability. Consequently, the safety assessment of the *B. licheniformis* T-1 strain was analyzed using a more complex model, Zebrafish [203].

Another model organism that is arousing considerable interest is *Galleria mellonella*. This organism is increasingly utilized to assess the pathogenicity and virulence of microorganisms [246], for the study of new or repurposed antibiotics [247,248], and for the evaluation of the safety and efficacy of probiotics [249].

Preliminary safety assessments of probiotics can also be conducted using mammalian animal models, such as mice. However, due to the associated management costs and ethical concerns surrounding the use of mammals, it is preferable to use the previously mentioned model organisms or fish directly. Among these, zebrafish is the most employed animal model for conducting preliminary safety analyses. Toxicity in zebrafish can be evaluated using both adult individuals, larvae, and embryos [250]. Acute toxicity experiments involve exposing fish to varying concentrations of the probiotic for a short duration to determine the lethal concentration at which a certain percentage of the fish perish (LD_50_ or LC_50_) [251,252]. Alternatively, the determination of LD_50_ was conducted directly on fish representing the probiotic hosts [173,182,188,189,203]. Conversely, chronic toxicity tests entail longer exposure periods, often lasting from days to several weeks [253]. In addition, changes in zebrafish behavior served as indicators of toxicity [223,227].

To evaluate probiotic toxicity, various methods can be employed, including mortality, biochemical markers, histological examinations, assessments of growth performance/body indices, and gene expression analysis, as described in subsequent sections.

### 3.4 Infection (challenge) or disease condition(s)

The effectiveness of a probiotic in improving health is often measured by exposing fish that have been given the probiotic to pathogens or stressful conditions. For this purpose, pollutants, dangerous chemicals, live microorganisms, dead microorganisms, or components thereof, and toxins can be used.

Different types of pollutants or toxic chemical compounds can be used for this purpose, such as heavy metals (lead, cadmium, mercury) [[7], [175],^176^,^180^,^204^,^205^,^209^,^232^] (Table 3 and Table S2). Fish can be exposed to heavy metals either through water contamination [[7], [204],^209^] or dietary intake [205] (Table S2). For instance, cadmium exposure was achieved by immersing fish in contaminated water containing 1–2 mg/mL of cadmium or by incorporating cadmium into their diet at similar concentrations [[7], [204],^205^,^209^]. Lead can be similarly introduced through water at concentrations of 0.05 mg/L, 0.5 mg/L, and 1 mg/L or via dietary intake (120 mg/kg and 240 mg/kg) [175,176,232]. Mercury, on the other hand, can be added to the diet at a concentration of 0.03 mg/L [180].

**Table 3:** Infection challenges: probiotics, infection/disease, methods of administration, and experimental output. Additional information on the experimental stetting and outputs were reported in Table S2.

Infection (challenge) or Disease condition(s)	Inoculum/Toxin/chemicals quantity	Administration	Host	Probiotic Strain(s)	Experimental output	References
*A. hydrophila*	0.1 mL 10^7^ CFU/mL	intramuscularly injected	*Oreochromis niloticus*	*Bacillus* sp. (ANSCI9, BFAR9, RM3, and RM10)	SR	[165]
*A. hydrophila*	10^7^ CFU/fish	intraperitoneally injected	*Labeo rohita*	*B. amyloliquefaciens* CCF7	MDA (liver) HP	[194]
*A. hydrophila*	50 μL 10^7^ CFU/mL	intraperitoneally injected	*Oreochromis mossambicus*	*B. licheniformis* Dahb1	SR	[202]
*A. hydrophila*	0.1 mL of 1 × 10^7^ CFU/mL	intraperitoneally injected	*Pangasius bocourti*	*B. aerius* B81e	SR HP	[210]
*A. hydrophila*	0.1 mL 2 × 10^7^ CFU/mL	intraperitoneally injected	*Oreochromis niloticus*	*B. licheniformis* HGA8B and *P. polymyxa* HGA4C	SR histopathology	[173]
*A. hydrophila*	10^5^ CFU/mL followed and 10^7^ CFU/mL after a week	Immersion	*Cirrhinus mrigala*	*B.* cereus SL1	SR histopathology	[206]
*A. hydrophila*	0.1 mL 10^6^ CFU/mL	intraperitoneally injected	*Lates calcarifer*	*B. subtilis* AAHM01	SR	[182]
*A. hydrophila*	10^6^ CFU/mL	intraperitoneally injected	*Labeo fimbriatus*	*B. subtilis* MTCC-121	SR HP	[183]
*A. hydrophila*	1 mL/100 g of weight 4 × 10^7^ CFU/mL	intraperitoneally injected	*Siniperca chuatsi*	*B. subtilis* 1-C-7	SR	[184]
*A. hydrophila*	0.2 mL 1 × 10^8^ CFU/mL	intraperitoneally injected	*Cyprinus carpio*	*B. velezensis* R-71003	Serum CFU count IGE	[193]
*A. hydrophila*	0.5 mL 2 × 10^6^ CFU/mL	intramuscularly injected	*Oreochromis niloticus*	*L. rhamnosus*	SR histopathology HP	[226]
*A. hydrophila*	0.02 mL 7.2 × 10^7^ CFU/mL	intraperitoneally injected	*Danio rerio*	*B. licheniformis* T-1	SR	[203]
*A. hydrophila*	0.2 mL 3.7 × 10^8^ CFU/mL	intraperitoneally injected	*Tor grypus*	*L. casei* PTCC1608 (ATCC 39392)	SR IGE	[229]
*A. hydrophila*	0.3 % weight, 1 × 10^7^ CFU/mL−1	orally exposed	*Ctenopharyngodon idella*	*B. subtilis* ch9	MDA (Serum, Intestine, and Liver) IGE Oxidative stress Anti-Ox	[177]
*A. hydrophila*	1 mL 1.6 × 10^7^	intraperitoneally injected	*Ctenopharyngodon idella*	*B. licheniformis* FA6	SR IGE	[200]
*A. hydrophila*	0.2 mL 1 × 10^8^ CFU/mL	intraperitoneally injected	*Oreochromis niloticus*	*B. velezensis* TPS3N, *B. subtilis* TPS4, *B. amyloliquefaciens* TPS17	SR	Kuebutornye et al., 2020
*A. hydrophila*	200 μL of 1 × 10^8^ CFU/mL	intraperitoneally injected	*Ctenopharyngodon idella*	*B. velezensis* BvL03	SR	[186]
*A. hydrophila* and *A. veronii*	100 μL of 1 × 10^6^ cfu/mL AhX040 150 μL of 1 × 10^9^ cfu/mL AvX005	intraperitoneally injected	*Ctenopharyngodon idella*	*B. amyloliquefaciens* X030	SR histopathology	[197]
*A. hydrophila X040* and *A. veronii X005*	0.1 mL of 1 × 10^6^ CFU/mL AhX040 & 0.15 mL of 1 × 10^9^ CFU/mL AvX005	intraperitoneally injected	*Cyprinus carpio*	*B. amyloliquefaciens* X030	SR IGE	[196]
*A. schubertii*	100 μL of 5 × 10^7^ CFU/mL	intraperitoneally injected	*Channa* spp.	*B. velezensis* WLYS23	SR	[192]
*A. veronii*	0.1 mL 10^7^ CFU/mL	intraperitoneally injected	*Ctenopharyngodon idella*	*B. velezensis* B8	SR	Wu et al., 2021
*A. veronii*	200 μL of 1 × 10^7^ CFU/mL	orally exposed	*Carassius auratus*	*B. velezensis* C-11, S-22, L-17 and S-14	SR, histopathology	[166]
*A. veronii*	10^7^ CFU/fish	intraperitoneally injected	*Oreochromis* spp.	*L. rhamnosus* GG	SR,	[225]
*E. tarda*	1 × 10^8^ CFU/mL	Immersion	*Danio rerio*	*B. subtilis* FI314, *B. vezelensis* FI436, and *B. pumilus* FI464	SR	Santos et al., 2021
*E. tarda*	0.1 mL 2 × 10^7^ CFU/mL	intraperitoneally injected	*Paralichthys olivaceus*	*B.* sp. SJ-10 (JCM 15709, KCCM 90078)	SR	[235]
*P. plecoglossicida*	5 μL 2.4 × 10^3^ CFU	intramuscularly injected	*Danio rerio*	*L. plantarum* E2	SR histopathology IGE	[220]
*S. agalactiae*	0.1 mL 10^7^ CFU/mL	intraperitoneally injected	*Oreochromis niloticus*	*L. plantarum* CR1T5	SR	[254]
*S. agalactiae*	10^7^ CFU/mL	intraperitoneally injected	*Oreochromis niloticus*	*L. plantarum* CR1T5	SR	[254]
*S. agalactiae*	0.1 mL 10^7^ CFU/mL	intraperitoneally injected	*Oreochromis niloticus*	*L. plantarum* CR1T5	SR	[216]
*S. iniae*	0.1 mL 7 × 10^3^ CFU/mL	intraperitoneally injected	*Oreochromis niloticus*	*B. mojavensis* B191 and *B. subtilis* MRS11	SR	[160]
*S. iniae*	100 μL 1 × 10^8^ CFU/mL	intraperitoneally injected	*Paralichthys olivaceus*	*B.* sp. SJ-10 and *L. plantarum* KCCM 11322	SR	[233]
*S. iniae*	50 μL of 10^5^ CFU/g of body weight.	intraperitoneally injected	*Oreochromis niloticus*	*B. safensis* NPUST1	SR	Wu et al., 2021
*S. iniae*	100 μL 1 × 10^8^ CFU/mL	intraperitoneally injected	*Paralichthys olivaceus*	*B.* sp. SJ-10	SR	[211]
*V. anguillarum*	2.7 × 10^7^ CFU/mL *V. anguillarum* LPS (100 μg/fish)	intraperitoneally injected	*Dicentrarchus labrax*	*B. velezensis* D-18	SR	[191]
*V. anguillarum*	0.1 mL of 5 × 10^7^ CFU/mL	intraperitoneally injected	*Anguilla japonica*	*B. subtilis* WB60	SR	[178]
*V. anguillarum* O1	7.5 × 10^4^ CFU/mL	intraperitoneally injected	*Etroplus suratensis*	*B. subtilis* MBTDCMFRI Ba37	SR	[179]
*V. harveyi*	0.1 mL of 1 × 10^7^ CFU/mL	intraperitoneally injected	*Epinephelus* sp.	*B. velezensis* K2	SR	[188]
VHSV (JF-09) - Virus	10^6^ TCID50/mL	orally exposed	*Paralichthys olivaceus*	*B. subtilis* DH2	cytopathic effects	[181]
*Y. ruckeri*	0.1 mL 10^7^ CFU/mL	intraperitoneally injected	*Oncorhynchus mykiss*	*L. rhamnosus* ATCC 7469	SR	[222]
Cd	1 mg/L	Contaminated water	*Cyprinus carpio*	*B. cereus*	MDA (Liver) Cd accumulation Anti-Ox	[7]
Cd	0.5 mg/L	Contaminated water	*Cyprinus carpio*	*B. coagulans* SCC-19	Cd accumulation tight junction gene expression levels of diamine oxidase and D-lactic acid	[209]
Cd	1 mg/L and 2 mg/L	Diet	*Carassius auratus gibelio*	*B. cereus*	MDA (Liver) Cd accumulation Anti-Ox	[205]
Cd	1 mg/L and 2 mg/L	Contaminated water	*Carassius auratus gibelio*	*B. cereus*	HP Cd accumulation IGE	[204]
Hg	0.03 mg/L	Diet	*Cyprinus carpio*	*B. subtilis*	HP IGE	[180]
Pb	120 mg/kg and 240 mg/kg	Diet	*Carassius auratus gibelio*	*B. subtilis*	HP Pb accumulation IGE Anti-Ox	[175]
Pb	0.05 mg/L, 0.5 mg/L and 1 mg/L	Waterborne	*Carassius auratus gibelio*	*B. subtilis*	HP Pb accumulation Anti-Ox	[176]
Pb	1 mg/L	Waterborne	*Cyprinus carpio*	*L. reuteri* P16	HP Pb accumulation IGE Anti-Ox	[232]
deltamethrin	15 μg/L	Waterborne	*Oreochromis niloticus*	*L. plantarum* L-137	HP IGE histopathology	[217]
PFBS	10 mg/L and 100 mg/L	Waterborne	*Danio rerio*	*L. rhamnosus* GG	Analysis of neurotransmitters, functions, inflammation and oxidative stress in brain	[223]
Type 2 diabetes mellitus	gradient hyper-glucose (50,100, 200 mM of glucose)	Waterborne gradient hyper-glucose accumulation methodology	*Danio rerio*	*L. rhamnosus* ATCC 53103	Blood glucose Histopathology IGE	[221]
Acute hypoxia/Exposure to Air	Oxygen levels decreased to 1 mg/L, air exposure stress	Netting fish out of the tank for 60 s	*Perca flavescens*	Fishery Prime™ (*B. subtilis, B. pumlis, B. amyloliqueficiens* and *B. licheniformis)*	Immune Response and Stress Tolerance	[167]
Alcoholic Liver Disease	0.5 % (v/v)	Waterborne	*Danio rerio*	*L. rhamnosus* GG	hepatic lipid accumulation, IGE gut permeability gene expression	[224]
Aflatoxin B_1_	0.5 mg/kg and1 mg/kg	Diet	*Liza ramada*	*L. acidophilus* ATCC 4356	Hepatorenal Functions, and Anti-Ox	[230]
Microcystin-LR	0, 2.2, and 22 μg/L	Exposure	*Danio rerio*	*L. rhamnosus*	bioaccumulation Neurotransmitters, Biochemical indicators hormone content	[227]

SR= Survival Rate; HP= Hematological paramenters; IGE= Immune-related or inflammatory-related gene expression; Anti-Ox = Antioxidant defenses.

Additionally, diverse diseases (T2DM, exposure to air/acute hypoxia, alcoholic liver disease) can be induced using chemicals or specific conditions during experiments [167,221,224] (Table 3 and Table S2). Other toxic compounds can be administered through exposure to contaminated water: PFBS (10 mg/L and 100 mg/L) [223], deltamethrin (15 μg/L) [217], and alcohol (0.5 % v/v) [224] (Table 3).

**Fig. 4:**
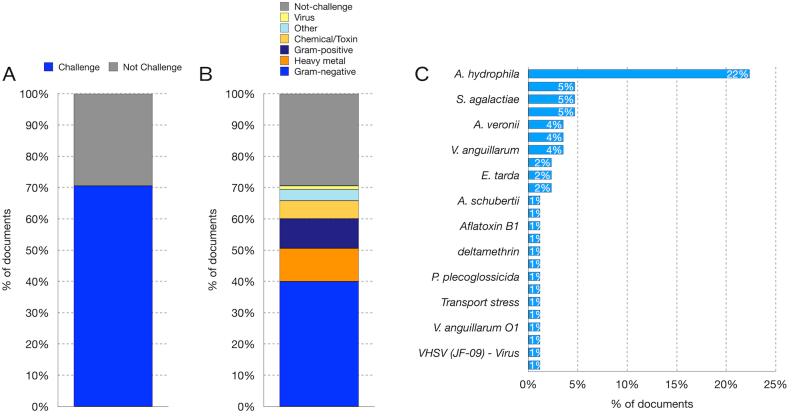
In vivo challenge experiments between a pathogen (or a disease) and the probiotic. A) Percentage of studies that included a challenge experiment. **B)** Types of pathogens or agents used for the challenge. **C)** Most common pathogenic bacteria used for the challenge.

Furthermore, fish can be challenged with live pathogenic microorganisms or with toxins such as LPS [191], aflatoxin B1 or Microcystin-LR [227,230] (Table 3). A challenge experiment was conducted in 70 % of the studies included in Table S2 (Fig. 4A). In most cases, the challenge involved only one bacterium, while only one case used a virus (Fig. 4A and B). Furthermore, 11 % of the documents focused on pathologies induced by heavy metal pollution (Fig. 4A and B, Table 3). The most frequently used pathogen was the Gram-negative bacterium *A. hydrophila*, followed by *S. iniae* and *S. agalactiae*, as well as *V. anguillarum* and *A. veronii* (Fig. 4B). Challenge experiments can also employ two pathogenic microorganisms; for example *A. hydrophila* and *A. veronii* (Fig. 4B).

Before conducting the infection experiment, some of the selected studies first conducted a preliminary experiment to determine the LD_50_ and calculate the bacterial load of the inoculum [173,188,189,203]. Most pathogenic bacteria were administered via intraperitoneal injection, involving the injection of 50–200 μL of bacterial suspension with loads ranging from 10^4^ CFU/mL to 10^9^ CFU/mL (Table 3 and Table S2). Typically, the fish were injected with a saline solution, such as PBS. Cumulative mortality (CM) and survival rate (SR) were determined to evaluate the effectiveness of the probiotic in mitigating the effect in the challenge experiments. It is important to note that other immunological and biochemical parameters were evaluated in these experiments. These parameters are discussed in the following sections.

### 3.5 Effect of probiotic on growth performance and host response

The host response to probiotic administration can be examined by considering molecular and biochemical markers, or by assessing growth performance (Table 4, Table S2). To measure growth performances, it is necessary to take anatomical measurements, including weight (W), length (L), and the weight of specific organs including the liver. These measurements are carried out before and after the experiments and it is important to consider both an experimental group (treated with the probiotic) and a control group. By combining this information, including the days of duration of the experiment and anatomical measurements, various parameters can be calculated such as absolute growth (AG), weight gain (or percent weight gain) (WG or PWG), specific growth rate (SGR), relative growth rate (RGR), daily growth index (DGI), increment in total length (ITL), body mass index (BMI), condition factor (CF), hepatosomatic index (HIS) and viscerosomatic index (VIS) (Table 4). In addition, to calculate body proximate composition (BPC), a standard protocol such as AOAC [255] must be followed. Additional parameters used to measure growth performance relate to indices calculated based on the quantity of feed supplied to the fish (feed intake, FI), including feed efficiency ratio (FER), feed conversion ratio (FCR), and protein efficiency ratio (FOR) (Table 4). Finally, cumulative mortality (CM) and survival rate (SR) were used to evaluate the mortality of fish reared on a probiotic-enriched diet compared to the control group (Table 4).

**Table 4:** Parameter used to assess the effect of probiotics on growth performance.

Parameter	Abbreviation	Formula
Initial numbers of fish	IN	–
Final numbers of fish	FN	–
Numbers of dead fish	NDF	–
Cumulative mortality	CM	100 × (NDF/IN)
Survival rate	SR	100 × ((FN/IN)-1)
Initial body weight	IBW (g)	–
Final body weight	FBW (g)	–
Absolute growth	AG (g)	FBW – IBW
Weight gain or Percent weight gain	WG or PWG (g days^−1^)	100 × ((FBW - IBW)/IBW)
Specific growth rate	SGR (g days^−1^)	100 × ((ln FBW - ln IBW)/days)
Relative Growth Rate	RGR	100 × ((FBW-IBW)/IBW)
Daily growth index	DGI (g/days)	100 × ((FBW)^1/3^ – (IBW)^1/3^)/duration
Length	L (cm)	**-**
Increment in total length	ITL (cm)	**-**
Body mass index	BMI (g cm^−2^)	FBW/L^2^
Condition factor	CF (g cm^−3^)	100 × (W/L^3^)
Hepatosomatic index	HIS	(g of liver × 100)/FBW or IBW
Viscerosomatic index	VSI	(g of viscera × 100)/FBW or IBW
Body proximate compositions	BPC	AOAC method [255]
Feed efficiency ratio	FER (g^−1^)	100 × (WG/g of consumed food)
Feed intake	FI (g)	g feed per animal per day
Feed conversion ratio	FCR	g of consumed food/(FBW-IBW)
Protein efficiency ratio	PER	WG/protein intake

However, evaluating only growth performance can be limiting and provide little information on the health status of the fish, especially concerning the immune response and inflammation. A simple method to evaluate the inflammatory process and the activity of the innate and adaptive immune systems in this context is real-time quantitative PCR (RT-qPCR) performed on RNA extracted from various tissues or organs taken from fish (kidney, gut or gut explant, spleen, liver, gill, brain) ([156,157,159,166,172,174,[176], [177], [178],^180^,^188^,^189^,^191^,^196^,^197^,^200^,^204^,^209^,^211^,^213^,^214^,^217^,^219^,[221], [222], [223], [224],^229^,[231], [232], [233]]; Yi et al., 2019; [157,160,167,173,182,193,199,206,207,220,227,228]). Among the genes typically used as markers for this type of analysis are interleukins and cytokines (IL-1β, IL-6, IL-8, IL-10, TNF-α, IL-4, IL-12, IL-13, IFN-γ, TGF-β1), enzymes involved in inflammation signaling (*cox2*), proteins associated with the innate immune response (membrane receptors like TLR2, TLR3, TLR4, TLR5, TLR7), antimicrobial enzymes/peptides, and other proteins such as MyD88, complement proteins (C3), immunoglobulins (IgM, IgD), lymphocyte markers (CD4-1 and CD4-2), stress proteins (HSP70 and HSP90), antioxidant enzymes (superoxide dismutase (SOD), catalase (CAT)), a superoxide-generating enzyme (NOX2), and proteins that regulate apoptosis following infection, such as caspases (CASP3, CASP2, CASP8, CASP9) (Table S2). This method can also be used to quantify the expression of junction proteins like ZO-1, ZO-2, claudin c, claudin 7a, and occluding [199,200,209].

As mentioned earlier, assessing the host response to the probiotic involves measuring enzymes or molecules (referred to as markers) in serum, blood, plasma, and specific organs. The blood, serum or plasma analyses typically cover general parameters such as hematocrit (HCT), total glucose (TGLU), total cholesterol (TCHO), total protein (Tpro), hemoglobin concentration (Hb), cortisol (stress hormone), triglycerides, albumin, creatinine, urea, or specific markers related to hepatic, renal, cerebral, or immune function, antioxidant activity, and the immune system. Markers used to measure liver function include bilirubin (total bilirubin (T-bilirubin), direct bilirubin (D-bilirubin)), alanine aminotransferase (ALT), aspartate aminotransferase (AST), alkaline phosphatase (AKP), and acid phosphatase (ACP). Other markers include blood urea nitrogen (BUN) for kidney function, lactic dehydrogenase (LDH) for measuring general tissue damage, and nitric oxide (NO) for assessing cardiovascular function. Crucially, oxidative stress measurement is closely related to overall health and the immune system. Various markers can be employed, including antioxidant enzymes such as SOD, myeloperoxidase (MPO), and antisuperoxide anion free radical activity (ASAFR).

Additional blood measurements focus on cell counts in this fluid, including white blood cell (WBC) count, red blood cell (RBC) count, total leukocyte count, and thrombocyte count. Immune cell activity assays like the phagocytic activity assay (PAA) or the proliferative response can be performed on immune cells such as peripheral blood leukocytes (PBLs) and peripheral blood monocytes (PBMs). Since the immune system's defense mechanisms involve the release of radicals against pathogens, respiratory burst activity (RBA) is considered an important parameter, along with the presence of lysozyme (LYM) and the activation of the alternative complement pathway (ACH50), both associated with the innate immune response, and the presence of IgM, associated with ongoing infectious processes. Similar measurements to those mentioned for serum, blood, and plasma can also be performed on specific organs, such as the liver, intestines, spleen, kidneys, and more. For instance, in the liver, various antioxidant enzymes and molecules associated with the oxidative state can be analyzed, including SOD, CAT, malonaldehyde (MDA), glutathione (GSH), and glutathione-associated enzymes (glutathione peroxidase (GSH-Px) and glutathione S-transferase (GST)). The spleen, a key organ in the immune system, can be harvested to isolate leukocytes and measure immune parameters (IgM, MCHII, B cell proliferation). The head kidney (analogous to the mammalian adrenal gland) can be used to measure parameters related to oxidative stress (SOD, RBA) and perform PAA tests with leukocytes. Skin mucus can also be utilized to measure some of these parameters, such as NO, LYM, IgM, AKP, CAT, SOD, and more.

In studies evaluating the efficacy of a probiotic in mitigating the effects of heavy metals (Cd and Pb), the accumulation of these metals was measured in different organs (gill, kidney, gut, liver, spleen, and muscle), as well as in serum or blood. In blood, the presence of the enzyme δ-aminolevulinic acid dehydratase (ALAD), typically inhibited by heavy metals but potentially more active in the presence of probiotics like *B. subtilis*, was quantified [176]. Furthermore, in these heavy metal studies, the efficacy of a probiotic was estimated by measuring antioxidant enzymes and oxidative stress in the liver (MDA, SOD, and CAT) and the gut (SOD, CAT, GSH-Px, ROS, MDA). In addition, biochemical parameters (AKP, IgM, LYM, NO, SOD, CAT, TPRO), as well as the activity of gut digestive enzymes (α-amylase, alkaline proteases, lipase, cellulase, and xylanase) and histomorphology can be measured to assess gut health.

### 3.6 Standardization of experimental conditions during in vivo esperiments

One of the major challenges in aquaculture experiments is the standardization of experimental conditions. First, the amount of probiotics included in the diet and the mode of administration should be standardized. Feed preparation should follow a clear and detailed protocol, with the probiotic content being monitored at the end of the process. The most accurate method to quantify the probiotic is by bacterial count (CFU/mL). Ensuring that other bacteria are not present in the feed or raw ingredients is crucial. However, bacteria phylogenetically close to the probiotic can appear almost identical when using cultural methods. For example, *Bacillus* species are common environmental contaminants and are often difficult to differentiate using only cultural methods or molecular methods like 16S rRNA gene sequencing. This issue can be addressed using a multilocus approach, which involves sequencing additional genes such as rpoD, gyrA, gyrB, and others [256,257]. Another molecular approach useful for distinguishing the probiotic from other genetically similar bacteria is genotyping through Random Amplified Polymorphic DNA-PCR (RAPD-PCR) [257]. This technique generates a specific fingerprint unique to each bacterial species. In addition, with the increasing accessibility of whole-genome sequencing (WGS), identifying probiotic-specific genes provides complementary phylogenetic markers.

It is essential to operate under sterile conditions during the preparation of probiotic-enriched feed, as environmental or feed-borne bacteria could influence the fish's response to the probiotic. Therefore, it is crucial to use selective media to monitor and quantify bacteria belonging to pathogenic genera, such as *Aeromonas*, *Vibrio*, and *Streptococcus*, that may be present in the feed [258]. Similarly, culture-independent molecular methods, such as gene-specific PCR, are useful in detecting the presence of genes associated with fish pathogens [[258], [259], [260], [261], [262]].

Further bacteriological controls, similar to those previously mentioned, should be performed on the water used in the experiments. Bacteria such as *Bacillus* and various fish pathogens can be present in the water. To prevent environmental bacteria from contaminating the experimental environment, systems for filtering and sterilizing the water should be implemented. Additionally, it is important to monitor the presence of algae or other microorganisms in the water that could affect fish health [263,264]. Moreover, controlling the physicochemical parameters of the water, as well as monitoring for pollutants, is essential, as these factors can influence both the performance of the probiotic and the health of the fish [265,266].

Other factors that may influence the performance of the probiotic include the health status of the fish used in the experiment and their colonizing microbiota. Therefore, it is important to monitor the hematological and immune parameters of the fish before starting the experiment. Several studies reviewed in this paper include analyses of various hematological and immune parameters. Similarly, monitoring the fish's microbiota before the experiments is crucial to identify any dysbiosis or other characteristics that could influence the outcomes. Challenges related to microbiota analysis may arise due to the inherent variability of bioinformatics and sequencing systems used in studying microbial communities through metagenomic methods. These limitations are discussed in the section of this review dedicated to microbiota.

## 4 Microbiome

One of the rapidly expanding branches of microbiology is the study of microbial communities that reside within an organism. Understanding the structure of microbial communities in the gut of fish, as well as comprehending how the community dynamically changes when a probiotic is introduced, is crucial for the development of effective probiotics. The methods primarily used to characterize the gut microbiota of a fish include both culture-based and culture-independent methods. These methods are used to evaluate the biodiversity of the total microbiota but also to understand whether a probiotic has colonized the host's intestine.

### 4.1 Cultural methods

The classical methods used to characterize the microbiota involve cultural techniques. In this approach, various types of media are utilized, including general selective media (incorporating antibiotics, bile salts, or other agents), media containing indicators (Table 2), minimal media containing specific carbon sources, and enriched media designed to support the growth of fastidious microorganisms. The combined use of these media can provide insights into bacterial load in terms of CFU/mL and offer a functional profile. For instance, plate count agar (PCA) was used to assess the amount of total aerobic bacteria in the gut of Nile Tilapia, while MRS was used to assess the amount of lactobacilli [232]. This last medium contains ammonium citrate and sodium acetate, which are selective agents effective against bacteria and molds. Additionally, the low pH and incubation in anaerobic conditions (5 % CO_2_) also limit the growth of organisms other than lactobacilli. This approach was used to demonstrate that the administration of a Pb-enriched diet caused a decrease in both the amount of total aerobic bacteria and the amount of lactobacilli in the gut of *C. carpio*. In contrast, feeding with *L. reuteri* P16 (10^8^ CFU g^−1^) caused an increase in the amount of both total bacteria and lactobacilli, showing that the probiotic survived in the gut of the fish whether they are fed with or without Pb.

### 4.2 Culture-independent methods: 16S rRNA metabarcoding

16S rRNA metabarcoding requires the extraction of total DNA from a sample, followed by the amplification of the 16S rRNA genes from the entire microbial community via PCR and subsequent sequencing. Once the sequences are obtained, bioinformatics tools are utilized to classify each sequence based on a database. The outcome is a table displaying the frequency of a particular sequence identification (reads).

Several studies on probiotics (Table S2) involve microbiota analysis through DNA metabarcoding. The 16S rRNA gene comprises nine variable regions, denoted as V1 to V9. The most used regions of the 16S rRNA gene for this purpose are the V3-4 regions [[7], [158], [174],^164^,^196^,^205^,^209^], but it is also possible to utilize the entire gene sequence (V1-V9) [213]. The Illumina MiSeq sequencing system is the most frequently used system for sequencing partial regions of the 16S rRNA gene (V3-V4) ([174]; Wang et al., 2022; [158,164,205,209]), although the same objective can be achieved using long sequencing system reads (PacBio) [196], which are generally used for sequencing the entire 16S rRNA gene [213].

Typically, the data processing workflow for this type of analysis consists of several steps, including: i) initial processing, which involves quality control, sequence filtering, demultiplexing, and more, ii) sequence clustering, iii) classification of the obtained reads at various taxonomic levels, ranging from kingdom to species. Several bioinformatics tools are currently available to assist in the management of 16S rRNA metabarcoding data. Among the most used pipeline tools are QIIME2, UPARSE, Mothur, VSEARCH, and DADA2 [[267], [268], [269], [270], [271]]. Additionally, especially for preliminary operations, various standalone algorithms are available or integrated into these pipelines: FastQC (https://www.bioinformatics.babraham.ac.uk/projects/fastqc/), which assesses the quality of obtained sequences; UCHIME (along with newer versions like UCHIME2 and uchime3_denovo) [272], used for identifying chimeric sequences; and the RDP classifier, a naive Bayesian classifier for sequence classification.

A crucial point of the computational process is the sequence clustering. For this purpose, OTUs (Taxonomic Operating Units) or ASVs (Amplicon Sequence Variants) are used. However, there are some differences between the two [273,274]. OTUs, generated by clustering sequences based on a chosen similarity threshold (most commonly 97 %) reflect a coarser notion of similarity than ASVs, which can resolve sequence differences of even a single nucleotide change [275]. An OTU contains several similar species of microbes grouped in a single unit. ASVs, also called exact sequence variants (ESVs) or zero-radius OTUs (zOTUs), can provide a more detailed picture of the diversity within a sample because they can be compared to a reference database with much higher resolution results. Finally, the sequence groups need to be classified. For this purpose, various databases are used, including Silva [276] and Green Genes (https://greengenes.secondgenome.com/) and various algorithms. The most common tool used to achieve this is the RDP classifier [277] which is set by choosing different threshold values ranging from 60 % to 80 % ([174]; Wang et al., 2022; [164,205,209]).

While 16S rRNA metabarcoding is a powerful technique for studying gut microbiota in fish, it has some limitations that should be considered such as the short read length, the use of different regions of the 16S rRNA gene, and the bias in PCR amplification. Generally, the platforms used for sequencing 16S rRNA genes produce readings with a maximum length of 300–400 bp, which can limit the accuracy of species identification. This issue can be improved using long-read sequencing using systems such as PacBio and Nanopore which results in sequences of >1000 bp. The PCR used to amplify the gene typically covers only two variable regions (V3 and V4) or even just one (V3), introducing variability in the results. PacBio and Nanopore long-read sequencing technologies offer several advantages over Illumina MiSeq for profiling gut microbiome composition. According to the literature, PacBio assigned 74.14 % of reads to the species level, compared to only 55.23 % for Illumina [278]. PacBio and Nanopore better estimate bacterial richness by capturing rare taxa missed by Illumina due to the higher sequencing depth required [279]. PacBio demonstrated a lower error rate than Nanopore technology [280]. Furthermore, different Nanopore flow cells exhibit different error rates: the R10.4 flow cell outperforms the R9.4.1 [280,281] However, Illumina can achieve higher throughput at a lower cost to obtain an equivalent number of sequences per sample [278]. While Illumina is cost-effective for high-throughput 16S sequencing, PacBio and Nanopore provide longer reads that enable more accurate species-level identification and richness estimation, especially for rare taxa. However, the bacterial community composition was similar between PacBio, Nanopore, and Illumina, with samples clustering by body site rather than sequencing platform [278,279,282]

Moreover, various algorithms offered by the same tool can be used for sequence classification, and the bioinformatics tools perform different calculations. It should be noted that different databases, as well as different versions of the same database, can be utilized for classification. The bioinformatics tools have advantages, disadvantages, and variable performance in terms of taxonomic classification [283]. QIIME2 is considered one of the better tools for taxonomic classification with a friendly interface and a flexible pipeline thanks to a plugin-based architecture that allows users to customize their analysis workflows [284]. DADA2 is recognized for its ability to resolve closely related sequences producing ASVs with high sensitivity [285]. However, DADA2, compared to methods like UPARSE, may produce a higher number of spurious ASVs [285].

Furthermore, the performance of the tools used varies greatly depending on the reference database used [283,286]. For example, when examining the gut microbiota of ruminants, no statistical differences were found between Mothur and QIIME (P > 0.05) in estimating the relative abundance of the most abundant genera (>10 %), either using SILVA or GreenGenes. However, differences were found in the least abundant genera (<10 %; P < 0.05) when using GreenGenes as the database. However, these differences were attenuated when SILVA was used as the reference database [286]. Hence, when analyzing an experiment, one should consider the strengths and weaknesses of each tool to choose the best one. For example, QIIME1 and Mothur are recommended when interested in rare and/or low-abundance taxa, QIIME2 is recommended if a rigorous filtering of artifacts is preferred, and would be appropriate to use at least two pipelines to evaluate the robustness of the results [287].

Overall, these issues limit taxonomic resolution. For these reasons, 16S rRNA metabarcoding data should be handled with caution and should be corroborated by other methods, including the culture methods described above or molecular techniques based on extraction of total DNA from gut samples followed by PCR [159].

### 4.3 Effect of probiotic on the fish microbiota

As mentioned earlier, several studies utilize 16S rRNA metabarcoding as a method to investigate changes in the intestinal microbiota of fish due to infections [164,196,213], heavy metal [[174], [205], [209], [288]], and probiotic administration (Table S2).

The most abundant phyla in *C. carpio* are Proteobacteria, Fusobacteria, and Firmicutes [[289], [290], [291]]. However, some studies have also identified other abundant phyla such as Bacteroidetes and Actinobacteria [291,292]. Similar microbiota colonizes the gut of *O. niloticus* [[293], [294]], and *C. auratus* [295].

Generally, the administration of Cd in carps has been found to significantly decrease the population of Proteobacteria and considerably increase Firmicutes, while the administration of Zn-enriched *B. cereus* can reduce changes in the composition of the intestinal microbiota [7]. Another study demonstrates that the abundance of Bacteroidetes increased with exposure to Cd, while the abundance of this phylum decreased when the probiotic *B. coagulans* SCC-19 was administered to fish [209]. In addition, Cd exposure affects intestinal microbiota diversity, and *B. coagulans* reverses this effect [209]. Infection with *Aeromonas* spp. changes microbiota composition, causing an increase in the abundance of Firmicutes and Bacteroidetes and a decrease in Proteobacteria [296]. Similar results were found in another study in which *Aeromonas*-infected carp showed an increase in the abundance of Fusobacteria, while the abundance of Proteobacteria the diversity and richness of microbiota were significantly decreased [297].

On the other hand, 16S rRNA metabarcoding provided in infection experiments suggests that the abundance of putative pathogenic *Aeromonas* was reduced in the probiotic-fed fish compared to the control groups [164,196,213]. In addition, there are evidence that the abundance of other putative pathogens, such as *Aromonas jandaei, Enterovibrio nigricans*, and *E. coralii*, was also reduced in probiotic-fed fish [213]. Furthermore, *Aeromonas* and *Roseomonas* were stimulated by mercury and blocked by a Se-*Bacillus*-enriched diet [174], while supplementation with Zn-enriched *B. cereus* decreased the abundance of pathogenic bacteria of the genus Flavobacterium [7]. Additionally, *B. coagulans* decreased the abundance of some pathogens (*Shewanella* and *Vibrio*) [209] in fish.

Finally, the abundance of potentially beneficial bacteria, such as *Lactobacillus* spp. and *Bacillus* spp., increased in probiotic-fed fish. For example, supplementation with *B. safensis* NPUST1 [213] as well as supplementation with *B. coagulans*, could reverse the altered intestinal microbiota diversity and increase the abundance the potentially beneficial bacteria belonging to the same genus of the potential probiotic added to the diet of fishes (*Bacillus*) and other potential beneficial bacteria, such as *Lactobacillus* spp [209,213].

## 5 Biomolecules produced by probiotics and their effects on fish

Bacteria produce many metabolites, some of which are beneficial to aquatic species. For example, Lactic Acid Bacteria (LAB) and *Bacillus* species produce a variety of metabolites, including organic acids, bacteriocins, amino acids, exopolysaccharides, and vitamins [298]. These bacteria produce various secondary metabolites, some of which exhibit antimicrobial activity [299]. Metabolites produced by probiotics that benefit aquatic animals are summarized in Table 5 and include short-chain fatty acids (SCFAs), bacteriocins, exopolysaccharides (EPS), neurotransmitters, and β-glucans. Furthermore, probiotics may also produce vitamins and amino acids that positively influence the health and growth of aquatic species.

**Table 5:** Biomolecules produced by probiotics and their effects on fish.

Biomolecules	Mechanisms
Short-chain fatty acids (SCFAs)	Improve growth performance
	Immunomodulatory effects
	Enhance disease resistance
	Modulate gut microbiota
Bacteriocins	Immunomodulatory effects
	Inhibit pathogenic bacteria
	Modulate gut microbiota
Exopolysaccharides (EPS)	Improve growth performance
	Enhance probiotic survival
	Improve gut barrier function
	Immunomodulatory effects
	Enhance probiotic adhesion to intestinal epithelial cells
	Modulate gut microbiota
	Improve antioxidant activity
Neurotransmitter	Improve growth performance
	Improve antioxidant activity
	Immunomodulatory effects
	Neurobehavioral benefits
β-glucans	Improve growth performance
	Immunomodulatory effects
Vitamins and amino acids	Supplement nutritional requirements of the host

SCFAs, such as acetate, propionate, and butyrate, are mainly produced by anaerobic microbiota thanks to the fermentation of carbohydrates in the intestine. SCFAs as feed additives improve growth performance, immune response, and disease resistance in farmed fish [300]. In addition, probiotics produce SCFAs. For example, multispecies probiotics containing bacteria like *Bacillus*, *Lactobacillus*, and *Pediococcus* increased the production of beneficial SCFAs and lactate [301].

Bacteriocins are ribosomally synthesized antimicrobial peptides produced by various bacteria, including probiotic strains such as LAB, and play a significant role in maintaining gut health and modulating microbial communities. Typically, bacteriocins kill the target cells thanks to the pore formation and the dissipation of cytosolic contents [302]. The action of bacteriocins is often strain-specific, allowing probiotic bacteria to outcompete pathogenic species in the gut [10,303,304]. Benefits of bacteriocins in aquaculture include the inhibition of pathogens, the modulation of gut microbiota, and the modulation of the host's immune response (enhancing the production of anti-inflammatory cytokines) [10,[302], [303], [304]]. In addition, bacteriocins are considered safe for consumption and can be used as natural preservatives in aquaculture, reducing the reliance on chemical antibiotics [304]. Bacteriocins produced by *Bacillus* and *Lactobacillus* species include lactocin, nisin, bacitracin, subtilin. *Lactobacillus* sp. MSU3IR has been shown to produce bacteriocins effective against fish bacterial pathogens like *V. harveyi* and *A. hydrophila* [305].

EPS are high-molecular-weight biopolymers secreted by microorganisms. They can form protective capsules around the bacteria, enhancing their survival and functionality in the gastrointestinal tract of aquatic animals [306,307]. EPS have been shown to possess immunomodulatory properties and improve gut health by promoting beneficial gut microbiota, thereby reducing the prevalence of pathogenic bacteria [[307], [308], [309]]. In addition, EPS improve growth performance and intestinal microbiota diversity in fish, such as juvenile turbot (*Scophthalmus maximus*) [308].

Probiotics in aquaculture can influence neurotransmitter production, such as serotonin, dopamine, acetylcholine, and gamma-aminobutyric acid (GABA) [310]. GABA dietary supplementation enhances growth performance and superoxide dismutase activity in *O. niloticus* [311,312]. Studies indicate that GABA can improve feed efficiency and overall growth rates [311,312]. In addition, GABA has been linked to the improvement of immune responses [312]. Bacteria, such as *L. plantarum* and *E. faecium*, have been identified as effective producers of GABA [313,314].

β-glucans, a type of soluble dietary fiber, have immunomodulatory properties that enhance the immune response and improve growth rates in aquatic species [[315], [316]]. For instance, studies have shown that dietary supplementation with β -glucans can significantly improve innate immune responses and disease resistance in fish like *O. niloticus* [317]. Probiotics can produce β-glucans. For example, *Pediococcus parvulus* 2.6 produces a 2-substituted (1,3)-β-D-glucan that show immunomodulatory proprieties (Pérez-Ramos et al., 2020).

In addition, probiotics produce vitamins and amino acids that enhance growth and health in aquatic species [318]. The probiotic strain *Pediococcus pentosaceus* L51, has demonstrated the ability to synthesize cobalamin (vitamin B12) [319]. Other vitamins produced by probiotic strains include riboflavin and folic acid [318]. Also, LAB produces amino acids [320].

## 6 Economic and managerial considerations in the use of probiotics in aquaculture

Using probiotics in aquaculture systems promotes fish health and enhances system performance. However, it is crucial to consider the economic and management implications of probiotic use in aquaculture. Therefore, the following paragraphs will discuss the economic implications, including the costs and benefits of probiotic application, regulatory affairs, and the environmental impact of probiotics, particularly concerning the sustainability of aquaculture systems.

### 6.1 Cost and benefits of the use of probiotics in aquaculture

The economic analysis of using probiotics in aquaculture includes several key aspects, such as initial investment costs, operational costs, and potential economic benefits. The implementation of production plants requires millions ($) of initial investments. Direct fixed capital (DFC) represents the fixed capital investment required for a complete process. It includes all the direct costs associated with plant implementation (TPIC, total plant direct cost), the indirect costs (TPDC, total plant indirect cost), and other associated costs such as contractors fee and contingency (CFC) necessary for the full capital investment in the plant (Table 6). Additional costs are related to the compliance of the probiotic and the production lines with safety and efficacy regulations. Other costs are associated with the purchase and installation of equipment needed to implement production lines including fermenters and equipment for downstream processing (e.g. drying) (Table 6). Once the initial investments have been made, the production of the probiotics involves some operating costs that include: the cost of raw materials (the reagents used in the production of probiotics), distribution costs (transport and storage), quality control costs, and maintenance of the equipment (Table 6). Table 6 summarizes the direct fixed capital (DFC), operating costs, and equipment purchase costs required to operate two different aquaculture plants: the first includes the fluidized bed drying and probiotic coating plant, the second is a plant using an optimized corn medium and spray drying [321,322].

**Table 6:** Summary of equipment cost, operating costs, and economic evaluation.

Parameter	Unit	Fluid bed drying and coating^a^	Optimized corn flour medium and spray-drying protective blends^b^
TPDC	Cost ($)	861000	1849000
TPIC	Cost ($)	516000	555000
CFC	Cost ($)	207000	361000
**DFC**	**Cost ($)**	**1584000**	2764000
Operating cost	Cost ($)	100000	3666000
Equipment purchase cost	Cost ($)	329350	1946000
**Total**	**Cost ($)**	**3597350**	**11141000**
Batch size	kg	326.91	3313.45
Payback time (PBT)	year	4.33	4.18
Cost basis annual	kg/year	34979	914513

DFC = Direct fixed capital; CFC= Contractors fee and contingency; TPIC = Total plant direct cost; TPDC = Total plant indirect cost.

a = [322].

b = [321].

Despite these initial and operating costs, the use of probiotics in aquaculture can produce significant economic benefits. For example, probiotics can improve growth rates and reproduction rates in aquatic species [323]. This leads to higher yields, which can significantly increase profitability. In addition, probiotics improve the health and immunity of aquatic organisms, which can lead to lower incidences of disease reducing the need for antibiotics and other expensive treatments, resulting in substantial savings [324]. Furthermore, it is important to analyze some market trends that favor products derived from fish fed with probiotics such as functional foods [325] and the reduction of antibiotic use. In conclusion, probiotics can favorably position aquaculture operations in the marketplace, potentially leading to higher sales prices and improved market access.

### 6.2 Sustainability

Probiotics in aquaculture have a positive environmental impact by improving water quality, reducing the need for antibiotics, enhancing growth and feed utilization, and minimizing waste and emissions [[326], [327], [328]]. Probiotics, particularly *Bacillus* species, play a significant role in improving water quality by influencing a wide range of parameters such as transparency, total dissolved solids, pH, conductivity, oxygen demand, dissolved oxygen, phosphates, and nitrogen species [329,330]. *Bacillus* can also be used for bioremediation of heavy metals and other pollutants [329,331]. Probiotics contribute to the decomposition of organic matter contributing to the recycling of nutrients [330,332]. Furthermore, a life cycle assessment (LCA) study showed that impact reductions generally offset the environmental impacts of probiotic production due to the lower consumption and reduced waste and emissions generation rates [328]. However, more research is needed to fully understand the impacts of probiotics on the environment and to address knowledge gaps, such as the selection and optimization of probiotics for specific aquaculture species, the development of effective delivery methods, and the assessment of potential ecological risks [326].

In the context of a more sustainable fish farming system, the effect that long-term exposure to probiotics may have on fish must also be considered. Long-term exposure of *C. auratus gibelio* to probiotics improves water quality and fish health [333]. Probiotics enhanced the degradation of ammonia and nitrate, improved biochemical parameters related to fish health, and reduced mortality after infection with *A. veronii* [333]. In another study, the long-term effects of three probiotics (*Lactobacillus buchneri*, *Lactobacillus fermentum*, and *Saccharomyces cerevisiae*, individually or in combination) on *Oncorhynchus mykiss* for 130 days were examined [334]. Indeed, some effects of probiotics were noted at the end of the experiments: after 130 days *S. cerevisiae* improved feed conversion and positively influenced the immune system, increasing the expression of TNF-α and IL-1β genes and reducing serum cholesterol [334]. Despite this information, long-term exposure to probiotics in aquaculture has not been studied extensively. Further studies are needed to understand the long-term effect of probiotics on fish and the environment. These studies should follow precise guidelines that specify the concentration of the probiotic, the duration of the experiments, the mode of administration of the probiotic, and the parameters to be measured.

#### 6.2.1 Side effects of probiotics on the environment

Most papers focus on the positive effects of probiotics on the sustainability of aquaculture facilities. However, relatively little is known about the potential adverse effects of probiotics, particularly concerning their impact on the environment and non-target organisms. Side effects include the disruption of natural microbial communities and the spread of antibiotic-resistant genes [[335], [336], [337]]. Also, it is important to consider that the depletion of natural communities and the excessive proliferation of probiotics could cause eutrophication and oxygen depletion in the water, harming other aquatic organisms.

It is important to verify that in the genome of a probiotic, there are no genes associated with antibiotic resistance, particularly the molecules used in the clinical field. In addition, it is important to check that probiotics do not produce toxins or other harmful substances, that could bioaccumulate up the food chain and negatively impact higher trophic levels, including humans consuming the aquaculture products. These factors should be preliminarily controlled using genomic methods, as discussed in this review.

In humans, side effects of probiotics include systemic infections, excessive immune stimulation in susceptible individuals, and impaired post-antibiotic microbiome reconstitution [336,338].

To mitigate these risks, it is important to carefully select probiotic strains that are safe for the environment, do not produce harmful substances, and are unlikely to persist or spread in the aquatic ecosystem. Proper containment and controlled application of probiotics are also crucial. Additionally, more long-term studies are needed to assess the ecological impacts of probiotics in aquaculture.

### 6.3 Regulatory landscape for probiotics in aquaculture

In Europe, the use of probiotics in aquaculture is regulated by several regulations: Regulation (EC) No. 1831/2003, Regulation (EC) No. 429/2008, Regulation (EU) 2019/6. Regulation No. 1831/2003 concerns additives for use in animal nutrition, including probiotics. Regulation (EC) No. 429/2008 provides detailed rules for the implementation of Regulation No. 1831/2003 as regards the preparation and submission of applications and the evaluation and authorization of feed additives. According to these regulations, the number of viable cells or spores expressed as CFU per gram shall be determined. They also indicate the safety controls that must be used to establish whether a microorganism can be used as a probiotic. These controls concern the production of virulence factors or toxins and the absence of antibiotic-resistance genes. The presence of toxins, virulence factors or other risks must be demonstrated, especially for strains belonging to a taxonomic group that includes members known to be able to produce toxins or other virulence factors. In addition, strains intended for use as additives must not present acquired antibiotic resistance genes unless it can be demonstrated that the resistance is the result of chromosomal mutations and is not transferable. Regulation (EU) 2019/6 may apply to certain probiotic products if are marketed as medicinal products. The European Food Safety Authority (EFSA) is responsible for evaluating the safety and efficacy of probiotics used in aquaculture through its Panel on Additives and Products or Substances used in Animal Feed (FEEDAP).

In the United States, the regulatory framework for probiotics in aquaculture is less well-defined than in Europe. Probiotics can fall into several regulatory categories depending on their intended use and claims: if a probiotic is intended to treat, cure, mitigate, or prevent disease in aquatic animals, it is considered a new animal drug and must be approved by the FDA's Center for Veterinary Medicine (CVM) before it can be marketed. If a probiotic is intended to affect the body structure or function of aquatic animals, but not to treat disease, it may be considered an animal feed ingredient and regulated by the FDA's Center for Food Safety and Applied Nutrition (CFSAN).

## 7 Innovation in aquaculture: smart aquaculture systems

The application of AI, including Machine Learning (ML) and other unsupervised algorithms, is an emerging field that aims to enhance the efficiency and sustainability of fish farming practices. Such computer methods can be useful for various purposes, including differentiating microorganisms that exhibit probiotic traits from others [339,340], analyzing data obtained from aquaculture experiments (for example concerning hematological parameters) [341], predicting the immunomodulatory effect of probiotics [342], monitoring fish health [343,344], and predicting the probiotic effect on the environment, such as the effect on water quality [345,346]. These systems can be integrated with other technological devices and connected to a single Internet of Things (IoT) network. ML has been used in conjunction with optical systems to automatically measure parameters, such as size and weight, but also to detect diseases [347]. Several reviews are available in the literature and provide a comprehensive overview of the applications of these systems in aquaculture [348,349].

## 8 Conclusion

Probiotics have become a focal point in aquaculture research, presenting a complex challenge in identifying their features, encompassing positive and negative characteristics. Positive attributes include the ability to interact with the host, enhancing nutritional and immune system aspects, and overall health improvement. Simultaneously, it is crucial to scrutinize the presence of toxins, virulence factors, antibiotic-resistance genes, and other potential threats to the host's and human health.

This review explores various methodologies employed in probiotic studies. Modern sequencing technologies enable the genomic analysis of microorganisms, aiding in the identification of genes coding for extracellular enzymes, secondary metabolites, and other positive probiotic characteristics. Genomic approaches also assist in detecting virulence factors, toxins, and antibiotic resistance-associated proteins, contributing to establishing a comprehensive safety and efficacy profile for potential probiotics.

Laboratory experiments, conducted both in vivo and in vitro, are pivotal. In vitro methods assess a probiotic's ability to adhere to cellular epithelia, produce extracellular enzymes, and exhibit bactericidal and biocontrol activity against pathogenic bacteria. Additionally, assays like hemolytic activity and antibiotic resistance screening help identify potentially hazardous traits.

In vivo testing is essential to assess several features of a probiotic, such as the safety, efficacy, and impact on the host immune system and overall health of the host. Challenge experiments, in which the host is infected with a pathogenic bacterium, demonstrate the biocontrol capabilities of a probiotic. Finally, culture-independent methods, such as 16S rRNA metabarcoding, offer insights into a probiotic's impact on the host's microbiota, correlating with the host's overall health and susceptibility to infectious diseases.

This review delves into these aspects, examining the methodologies employed and the expected outcomes.

## CRediT authorship contribution statement

**Matteo Calcagnile:** Writing – review & editing, Writing – original draft, Conceptualization. **Salvatore Maurizio Tredici:** Writing – review & editing, Writing – original draft. **Pietro Alifano:** Writing – review & editing, Writing – original draft.

## Funding

The work was partially supported by fish RISE project (PON 2014/20 ARS01_01053).

## Declaration of competing interest

The authors declare that they have no known competing financial interests or personal relationships that could have appeared to influence the work reported in this paper.
